# Supplementary material for: Worldwide Late Pleistocene and Early Holocene population declines in extant megafauna are associated with Homo sapiens expansion rather than climate change
Source: Nat Commun. 2023 Nov 24;14:7679. doi: 10.1038/s41467-023-43426-5 (PMC10667484; doi:10.1038/s41467-023-43426-5)
Supplement: Supplementary file 1 — Supplementary Information [file 41467_2023_43426_MOESM1_ESM.pdf]

Supplementary Information

Contents

Supplementary Note 1: Statistical modeling....... 1
- Mutation rate and generation time model (Supplementary Fig. 1)....... 1
- Mass-based model across the full time span (Fig. 1b)....... 1
- Decline severity model across the full time span (Fig. 1c)....... 2
- Climate-based predictive models (Fig. 2 and Supplementary Fig. 11)....... 3
- Climate and human-based explanatory models (Fig. 3 and Supplementary Fig. 13)....... 5
- Estimation of megafauna census size, biomass and energy turnover (Fig. 4 and Supplementary Fig. 15)....... 7

Supplementary Note 2: Breakpoint analysis....... 10

Supplementary Note 3: Phylogeny effect....... 12

Supplementary Note 4: Human-megafauna PSMC trajectory relationship....... 13

Supplementary Note 5: Alternative quantification of past megafauna census sizes....... 14

Supplementary Note 6: Comparison of interglacial population dynamics....... 18

Supplementary Figures....... 19

Supplementary Tables....... 37

References....... 47

Supplementary Note 1: Statistical modeling

Mutation rate and generation time model (Supplementary Fig. 1)

The conversion of the PSMC output to effective population sizes and time intervals in years requires knowledge of the per generation mutation rates and generation times[1]. While generation times are easily obtained from literature (Supplementary Data 1), mutation rates are generally not available for the majority of species. However, we can use the known relationship between mutation rates and generation times in mammals to predict the mutation rate for species where these data are missing. To fit this model, we used the empirically estimated mutation rates and average parental ages (generation times) from 61 sequenced mammalian families[2]. The model is represented as follows:

$$M_i \sim \Sigma N(\mu_i, \sigma)$$
$$\mu_i = a + bG_i$$
$$a \sim N(0, 0.1)$$
$$b \sim N(0, 0.1)$$
$$\sigma \sim exp(0.001),$$

where *M* is the observed per generation mutation rate in a mammalian family and *G* is generation time. The mutation rate *M*, as well as prior distributions for the intercept (*a*) and slope (*b*) of the relationship are assumed to be normally (*N*) distributed, while the model error σ is assumed exponentially distributed (*exp*). We fitted the model with the *G* predictor either on a natural scale or log-transformed. Both models have similar predictive accuracy, as demonstrated by the similarity of their leave-one-out cross-validation log-score - 1103.47 ± 5.86 and 1103.46 ± 5.47 for the model with and without the log-transformation of *G*, respectively. However, as the validation log-score is on average higher for the model with the log-transformed *G* predictor, we use this model to predict mutation rates for species where these data are unavailable. Specifically, we use the posterior distributions of the *a* and *b* coefficients to estimate the *M* distributions for each species, and use the medians of these distributions when transforming the PSMC output.

Mass-based model across the full time span (Fig. 1b)

Here, we model the change in effective population size (N_e) of megafauna species as a function of time (*t*) and species' adult mass (*m*). Prior to inference, both the N_e and *t* values were $\log_{10}$-transformed. The model allows for varying intercepts and slopes across species, and incorporates the effect of species adult mass as follows:

$$y_i \sim N(\mu_i, \sigma)$$
$$\mu_i = a_{j[i]} + b_{j[i]} t_i$$
$$a_j \sim N(\gamma_j, \zeta)$$
$$b_j \sim N(\delta_j, \kappa)$$

$$\gamma_j = g_0 + g_1 m_j$$
$$\delta_j = h_0 + h_1 m_j$$
$$g_0 \sim N(5, 1)$$
$$(g_1, h_0, h_1) \sim N(0, 1)$$
$$(\sigma, \zeta, \kappa) \sim exp(1),$$

where y_i is an N_e value at a specific time in the past t_i. The variable y_i is modelled as normally distributed with mean μ_i and standard deviation σ. The second line describes the global linear regression model of N_e as explained by t_i, used to infer species-specific slopes (a_j) and intercepts (b_j), where the subscript j indicates a specific megafauna species. Species-specific slopes and intercepts were both modelled as response variables of a nested linear regression with adult mass as an explanatory variable (m_j). A normal distribution with mean γ_j (δ_j) and standard deviation ζ (κ) was assumed for the species-specific slope (intercept) of the nested models. The coefficients of the nested models ($g_{\{0,1\}}$ and $h_{\{0,1\}}$) were subscripted with 0 for intercepts and 1 for slopes, and assigned normal prior distributions. Standard deviations of variables (σ, ζ and κ) were assigned an exponentially distributed prior.

Decline severity model across the full time span (Fig. 1c)

We define decline severity as

$$D = 1 - \frac{N_e^{MIN}}{N_e^{MAX}},$$

where N_e^{MIN} and N_e^{MAX} are the minimum and maximum effective population sizes experienced by a species during the full timeframe of the PSMC-estimated population size trajectory, respectively. The Bayesian framework of the generalised linear model is as follows:

$$D_i \sim B\left(Logit^{-1}(\mu_i), \sigma\right)$$
$$\mu_i = a + b_1 m_i + b_2 t_i^{MIN} + b_3 t_i^{MAX}$$
$$(a, b_1, b_2, b_3) \sim N(0, 1)$$
$$\sigma \sim HalfCauchy(0.1),$$

where the response variable D is assumed to be Beta-distributed (B) with mean value μ and standard deviation σ, and a logit link function. The predictors of D are species' adult mass (m), time when a species achieved the lowest population size (t^{MIN}) and time when a species achieved the highest population size (t^{MAX}), with b_1, b_2, b_3, as the corresponding coefficients and a as the intercept of the regression model. The predictor variables were transformed to a scale with mean 0 standard deviation of 1 (Z-score). This was done to simplify computation and achieve comparability between the estimated b coefficients. We assume a normally distributed prior (N) for the intercept and coefficients, and a Half-Cauchy (*HalfCauchy*) prior for σ.

Climate-based predictive models (Fig. 2 and Supplementary Fig. 11)

Climate-based models assessed the relationship between climatic variables and population size for the period between the present and 742,419 years ago (the time span for which estimates of the climatic variables are available). Here, we used N_e estimates from time intervals older than 100,000 years for fitting the models, while N_e values between the present and 100 kya were predicted using the fitted models, and compared to the observed PSMC-inferred N_e estimates. Prior to prediction, we discretised this time frame into four equally-sized 25,000-year time windows to account for between-species differences in sizes of time windows and facilitate the comparison of model performance between time points. The goal of this modelling approach is to assess the level at which the relationship between climate fluctuations and population size prior to 100 kya can explain population size fluctuations of the recent past (between 100 kya and the present).

We also note that in the following models we assume weak prior distributions (e.g. centered at 0 if we consider slopes of relationships), as well as adopt the strategy of partially pooled modelling, where we assume a cross-species pooled error distribution for every model. These considerations make it such that the need for explicit correction of multiple testing is alleviated.

We modelled the relationship between mean annual temperature (and/or mean annual precipitation) and population size using two different models. In model explanations below, we use the temperature parameter (T) to demonstrate the relationship between a climatic predictor and N_e; however, please note that all models were also constructed with mean annual precipitation as predictor, as well as all combinations of temperature and precipitation as predictors. Firstly, we implemented a basic linear regression

$$N_e(T) = a + bT,$$

where a and b represent the intercept and slope of the relationship, while T is the average temperature over the focal time interval for which we have an estimate of population size N_e. While simple, this model does not necessarily reflect biological reality due to the assumption of a linearity between population size and temperature.

To implement a more biologically realistic model, we assume that each species has an optimal temperature (T_{opt}) at which N_e is maximised. As temperature deviates from T_{opt} in either direction, we expect a decrease in population size. Such a relationship can be described by a quadratic function

$$N_e(T) = a - b(T - c)^2,$$

with the requirement that $b<0$. Taking the first derivative and setting the function to 0, it can be shown that the maximum of this function is c, *i.e.* $T_{opt}=c$.

In addition to considering temperature of the focal time interval, we also consider a temperature lag parameter L, defined as the average temperature of the preceding time interval.

The Bayesian frameworks are as follows:

$$y_i \sim N(\mu_i, \sigma)$$
$$\mu_i = a_{j[i]} + b_{j[i]} T_i$$

$$a_j \sim N(5, 1)$$
$$b_j \sim N(0, 1)$$
$$\sigma \sim exp(1),$$

for the linear model ($linT$);

$$y_i \sim N(\mu_i, \sigma)$$
$$\mu_i = a_{j[i]} - b_{j[i]}(T_i - c_{j[i]})^2$$
$$a_j \sim N(5, 1)$$
$$b_j \sim HalfCauchy(1)$$
$$c_j \sim N(0, 1)$$
$$\sigma \sim exp(1),$$

for the quadratic model ($quadT$);

$$y_i \sim N(\mu_i, \sigma)$$
$$\mu_i = a_{j[i]} + b_{j[i]}T_i + c_{j[i]}L_i$$
$$a_j \sim N(5, 1)$$
$$b_j \sim N(0.5, 1)$$
$$c_j \sim N(0, 1)$$
$$\sigma \sim exp(1),$$

for the linear model with lag ($linT + L$);

$$y_i \sim N(\mu_i, \sigma)$$
$$\mu_i = a_{j[i]} - b_{j[i]}(T_i - c_{j[i]})^2 - d_{j[i]}(L_i - e_{j[i]})^2$$
$$a_j \sim N(5, 1)$$
$$(b_j, d_j) \sim HalfCauchy(1)$$
$$(c_j, e_j) \sim N(0, 1)$$
$$\sigma \sim exp(1),$$

for the quadratic model with lag ($quadT + L$). Priors are defined as normally (*N*), exponentially (*exp*) or Half-Cauchy (*HalfCauchy*) distributed. All model parameters were estimated for each species *j* separately, apart from the model error σ, which is a pooled estimate across species.

Climate and human-based explanatory models (Fig. 3 and Supplementary Fig. 13)

Here, we were interested in the explanatory power of climate and human impact on past population sizes of megafauna. To model the impact of climate we consider the linear and quadratic models from the previous section, in combination with human impact. The first human impact parameter we consider is the probability of human presence (*p*), which was constructed in the following way. For each species, we consider the human arrival range based on the ecological realm of that species[3] or, in the case of the Afrotropic realm, the timeframe of expansion of *H. sapiens* throughout the Afrotropic realm (Supplementary Table 3). We assign a value of *p* between 0 and 1 to each time window for which we have an estimate of the species population size. Specifically, for time windows prior to the human arrival range, p is assigned the value of 0. Windows that overlap or postcede the human arrival range are assigned a value larger than 0, depending on the span of the human arrival range and overlap of this range with the focal time window

$$p = 0 \quad \text{if} \quad t_l > a_u$$
$$p = 1 \quad \text{if} \quad t_u < a_l \quad \text{or} \quad t_l < a_l$$
$$p = \frac{a_u - t_l}{a_u - a_l} \quad \text{otherwise,}$$

where t_l and t_u are the lower and upper bounds of the focal time window, respectively, and a_l and a_u are the lower and upper bounds of the human arrival range. Supplementary Table 11 shows an example calculation of *p* for five consecutive 25-ky time windows given a human arrival range between 40 and 95 kya.

The p parameter can be thought of as cumulative human impact over time that reaches its maximum value of 1 in the time window that overlaps the lower bound of the human arrival range, and maintains this value throughout subsequent windows, towards present time. In that way, it is a conservative estimate of human impact, which, in reality, continued to increase post human arrival.

The Bayesian frameworks for these models are represented as:

$$y_i \sim N(\mu_i, \sigma)$$
$$\mu_i = a_{j[i]} + b_{j[i]} p_i$$
$$a_j \sim N(5, 1)$$
$$b_j \sim N(0, 1)$$
$$\sigma \sim exp(1),$$

for the model with only human presence as a predictor (pH);

$$y_i \sim N(\mu_i, \sigma)$$
$$\mu_i = a_{j[i]} + b_{j[i]} T_i + c_{j[i]} p_i$$
$$a_j \sim N(5, 1)$$
$$(b_j, c_j) \sim N(0, 1)$$

$$\sigma \sim exp(1),$$

for the linear temperature and linear human impact model ($linT + pH$), and

$$y_i \sim N(\mu, \sigma)$$
$$\mu_i = a_{j[i]} - b_{j[i]}(T_i - c_{j[i]})^2 + d_{j[i]}p_i$$
$$a_i \sim N(5, 1)$$
$$b_j \sim HalfCauchy(1)$$
$$(c_j, d_j) \sim N(0, 1)$$
$$\sigma \sim exp(1),$$

for the quadratic temperature and linear human impact model ($quadT + pH$). For comparison, we also run the models with only the climate predictors (temperature-only, precipitation-only or a combination of the two) or only the human predictor, as well as models with the climate lag parameter L.

We then considered a second type of human impact model, where humans are expected to start affecting megafauna population size after their earliest arrival date (a_u) to the biogeographic realm (Supplementary Table 3), while prior to human arrival, we assume a constant population size. Such a model can be written as

$$N_e(H, t) = a + Hbt,$$

where the predictor H takes the value of 1 or 0, depending on whether or not there is overlap between human arrival range and the focal time window, respectively. Additionally, H takes the value of 1 for all windows that postcede human arrival. Further, a is the constant population size prior to human arrival, and the expression Hbt describes the time-dependent population size change following human arrival. The predictor t was transformed such that it reflects the timespan of human-megafauna interaction (i.e. $t = 0$ when $H = 0$, $t > 0$ when $H = 1$, and t reaches maximum value at present time). We also considered two models with a non-linear population size change post-arrival. Firstly, we consider a model with exponential impact

$$N_e(H, t) = (1 - H)a + Hae^{rt},$$

with H and a as in the previous model and r as the rate of population size change following human arrival. Secondly, we consider a logistic impact model

$$N_e(H, t) = (1 - H)\frac{a}{1+c} + \frac{Ha}{1+ce^{rt}},$$

with H as in the previous model and the expression $\frac{a}{1+ce^{rt}}$ describing the logistic change in megafauna population size following human arrival. Specifically, a and c are constants determining the intercept of the logistic expression ($\frac{a}{1+c}$) and r is the rate of population size change.

The Bayesian frameworks for these models are represented as:

$$y_i \sim N(\mu_i, \sigma)$$
$$\mu_i = (1 - H_i)a_{j[i]} + H_i b_{j[i]} t_i$$
$$a_j \sim N(5, 1)$$
$$b_j \sim N(0, 1)$$
$$\sigma \sim exp(1),$$

for the linear human impact model ($linH$),

$$y_i \sim N(\mu_i, \sigma)$$
$$\mu_i = (1 - H_i)a_{j[i]} + H_i a_{j[i]} e^{r_{j[i]} t_i}$$
$$a_j \sim N(5, 1)$$
$$r_j \sim N(-1, 3)$$
$$\sigma \sim exp(1),$$

for the exponential human impact model ($expH$), and

$$y_i \sim N(\mu_i, \sigma)$$
$$\mu_i = (1 - H_i)\frac{a_{j[i]}}{1+c_{j[i]}} + \frac{H_i a_{j[i]}}{1+c_{j[i]} e^{r_{j[i]} t_i}}$$
$$a_j \sim N(5, 1)$$
$$c_j \sim HalfCauchy(0.1)$$
$$r_j \sim N(5, 3)$$
$$\sigma \sim exp(1),$$

for the logistic human impact model ($logH$). Priors are defined as normally (*N*), exponentially (*exp*) or Half-Cauchy (*HalfCauchy*) distributed. We also ran these models in combination with four of the best-fitting models with climate predictors. In total, we tested and compared 32 models (Supplementary Table 10 and Supplementary Fig. 12). All model parameters were estimated for each species *j* separately, apart from the model error σ, which is a pooled estimate across species.

Estimation of megafauna census size, biomass and energy turnover (Fig. 4 and Supplementary Fig. 15)

To estimate census sizes (N_c) of megafauna for different time periods we first calculated the ratio (r) of the Holocene N_e (as a proxy for the current effective size of a species) and their current IUCN census size for every species with available IUCN N_c estimates in our dataset (99 species in total, as of September, 2022). The r ratio of species whose Holocene N_e was greater than their current IUCN census (16/99 species in total) was set to the median r ratio of species with $N_e < N_c$ (median $r = 0.114$; after exclusion of $N_e > N_c$ species). This was done as species with $N_e > N_c$ have likely undergone the most severe reductions

in census size during the recent past, resulting in the uncoupling of the N_e~N_c relationship. The r ratio of these species is thus unsuitable for transforming their N_e into estimates of N_c. Similarly, the r ratios of species with unavailable IUCN census estimates were set to the same median r value. We then multiplied N_e values with species-specific $1/r$ estimates to transform N_e into estimates of N_c across different past time points (baseline period: 100-742 kya; four 25,000-year periods across the last 100,000 years). For the current time point, the N_c was set to the IUCN census estimate, or, in the case of species without IUCN census estimates, the current N_c was estimated by multiplying their Holocene N_e with $1/r_c$, where r_c (= 0.155) is the median ratio of Holocene N_e and current IUCN census estimates calculated using the full set of 99 species for which current IUCN N_c estimates were available. Thus, r_c also reflects the severe recent reductions of census sizes and uncoupling of the N_e~N_c relationship.

We then summed N_c values across the 139 species in our dataset to get the total megafauna census size for each time period (Fig. 4a). Additionally, the N_c values were multiplied by the corresponding adult mass of each species (as reported by PHYLACINE[4]) or daily metabolic rate from Pedersen *et al.* (2023)[5], followed by across-species summation to get total biomass and energy turnover estimates.

In order to obtain N_c values, biomass and energy turnover for species missing in our dataset (i.e. extant megafauna species for which suitable genomic data were unavailable, as well as extinct megafauna species), we first used the PHYLACINE database to extract species information of all extant and extinct terrestrial mammals above or equal to 22 kg (corresponding to the mass of the smallest megafauna species included in our original dataset). We then filtered out species that were included in the original 139-species dataset, which left us with 318 missing megafauna species (121 extant and 197 extinct megafauna species). For each of these species we extracted adult mass data using PHYLACINE and daily metabolic rates from Pedersen *et al.* (2023)[5]. Then, using the original 139-species dataset we fitted the linear model

$$y_i \sim N(\mu_i, \sigma)$$
$$\mu_i = a + bm_i$$
$$a \sim N(5, 1)$$
$$b \sim N(0, 1)$$
$$\sigma \sim exp(1),$$

where y is the N_c value of a species and m is the adult mass. We assumed a normal distribution (N) for the N_c values (y) and the priors of the intercept (a) and slope (b) of the relationship, and an exponential (exp) prior for the model error σ. Both N_c and m were $\log_{10}$-transformed prior to model fitting. We fit the model using two sets of N_c estimates: baseline (100-742 kya) N_c estimates and current N_c estimates of the original 139-species dataset. We used the adult mass estimates from PHYLACINE and coefficients of the model fitted using baseline N_c to predict N_c values of extinct and missing extant species during the baseline period, while the model fitted using current N_c was used to predict current N_c of missing extant species. Specifically, for each extinct and missing extant species we sampled the posterior distributions of coefficients to create a 1,000-sample distribution of N_c values. We then summed the species-specific N_c distributions to create a 1,000-sample distribution of the sum of N_c values. The medians of these distributions (*i.e.* the N_c-sum distribution of extinct species for the baseline period, and N_c-sum distributions of missing extant species for the baseline and current periods) were used to calculate the relative contribution of different megafauna categories (extinct species, missing extant species and species

in the original 139-species dataset) to the total megafauna N_c during the baseline and current periods (Fig. 4b). Similarly to before, per-species N_c values of extinct and missing extant species were also multiplied by the corresponding adult mass value (as reported by PHYLACINE) or daily metabolic rate[5] to obtain the relative contribution of different megafauna categories to the total biomass and energy turnover during the baseline and current periods (Fig. 4b).

Lastly, we attempted to alleviate the likely underestimation of past megafauna census sizes, given that all estimates of past N_c were dependent on current IUCN N_c estimates of species that underwent population contractions in the very recent past. To do this, we repeated all the analyses above using a modified r ratio (r_{mod}) for estimating species-specific N_c during past time points (baseline period: 100-742 kya; four 25,000-year periods across the last 100,00 years), while N_c estimates for the current period were kept identical (*i.e.* as estimated by the previous analysis). The r_{mod} ratio was calculated as

$$r_{mod} = cr,$$

where c in a constant and r corresponds to the species-specific r ratios of the previous analyses. The constant c is a function of species census sizes prior to extremely severe N_c reductions of the recent past. The closest estimates of such census sizes are known from 19th and early 20th century records of the American bison[6] and African elephant[7], with census populations of 30 and 27 million, respectively. Given these historical census estimates, the r ratio for the bison and elephant would be 0.00017 and 0.00028, respectively (using Holocene N_e), while given current IUCN estimates, the r ratio of these species is estimated to be 0.408 and 0.012, respectively. On average, the r ratio based on historical census sizes is thus only 0.0118× that of the r ratio based on current IUCN N_c estimates. We therefore set c = 0.0118, calculated the r_{mod} ratio for each species and used it to re-estimate total census size, biomass and energy turnover of megafauna during past time points (Supplementary Fig. 15).

Supplementary Note 2: Breakpoint analysis

To obtain the breakpoint range presented in Fig. 1a we divided species into six biogeographical realms (Afrotropic, Palearctic, Indomalaya, Australasia, Nearctic and Neotropic). The classification of species is based on the percent overlap of the species' present natural range (estimated from PHYLACINE[4]) with each of the realm-specific geographical ranges. To ensure accurate classification, we only included species for which we had an exact match between the binomial naming convention of the PHYLACINE database and the curated genomics dataset (Supplementary Data 2). Additionally, if multiple subspecies were available in the genomics dataset, we chose a single representative of the species to match it with the PHYLACINE naming convention (*e.g.*, *Giraffa camelopardalis tipplekirschi* from the genomics dataset was matched to *Giraffa camelopardalis* from the PHYLACINE database, while the rest of the *Giraffa* subspecies were discarded). This filtering procedure left us with 100 species available for analysis (Supplementary Data 2).

We classified each of the 100 species according to the largest percent overlap of their present natural range with the realm-specific geographical ranges (Supplementary Data 2) - therefore, for each species we designated a *majority* realm, where most of the species' present natural range is located. Using the "segmented" library[8] implemented for the R programming language, we estimated the time breakpoint (and standard error) for each realm by fitting a piecewise linear model to the PSMC-estimated population size dynamics of species with the corresponding majority realm. In effect, we estimated two time periods with varying intensities of megafauna population decline for each realm (Supplementary Table 1). The breakpoint range presented in Fig. 1a was set between 31,999 and 75,682 years ago, corresponding to the most extreme values of breakpoint ranges across realms. We similarly calculated breakpoints by assigning species according to their biome (Supplementary Table 4) and human biogeography (Supplementary Table 5).

When considering realm-specific breakpoints (Supplementary Table 1), the Indomalaya and Afrotropics have the oldest breakpoints, followed by Australsia, Neotropics, Palearctic and finally, Nearctic. Furthermore, the Nearctic, with its youngest estimated breakpoint, had the most negative slope for the time period after the breakpoint (i.e. between the breakpoint and present time), while regions with older breakpoints had significantly negative, but less severe slopes. The slopes estimated for the period prior to the breakpoint were lower in magnitude, and generally negative, except in the cases of Australasia (significantly positive slope prior to the breakpoint), Neotropics and Nearctic (non-significant slopes).

When considering biome-specific breakpoints (Supplementary Table 4), we observed the oldest breakpoint for the polar biome, that is more than double the age of the next oldest biome (the tropical biome). The cold biome had an intermediary breakpoint, while the arid and temperate biomes had the youngest breakpoints. We again observe that biomes with younger breakpoints have a more severe, negative slope, compared to biomes with older breakpoints. Additionally, both time periods had a significantly negative slope across all biomes, with the exception of the polar realm (non-significant slope). Furthermore, more severe slopes were estimated for the time period after the breakpoint.

When considering human biogeography regions (Supplementary Table 5), we estimated the youngest breakpoints for regions where archaic *Homo* species arrived early, or where *Homo sapiens* was the only *Homo* species present, followed by regions where archaic *Homo* species arrived late, regions where the *Homo* genus evolved, and finally archaic-peripheral regions. We again detect generally negative slopes across both time periods, with the exception of before-breakpoint periods for archaic-peripheral and *H. sapiens*-only regions (non-significant slopes). Slopes of after-breakpoint

periods are again more severe and show a dependency on breakpoint timing. Together, these results show that megafauna decline proceeded more severely during periods closer to present time, especially within non-Afrotropic regions with suitable human-inhabitable regions such as the temperate biome. However, while the Nearctic and Neotropics have some of the youngest breakpoints, their lack of correspondence with human arrival times into these regions (Supplementary Table 3) points to an unrelated environmental cause for the onset of megafauna decline in these regions.

As species often occupy multiple realms, biomes and human biogeography regions (Supplementary Data 2), we calculated breakpoints and slopes when including species with a proportion of their present natural range within a specific geographic region (Supplementary Tables 6-8). We generally observed that breakpoint estimates that include species with >10% or >25% of their present natural range within a specific region, are often significantly different from estimates based on species that have >50% of their range within the region. This is likely due to the fact that >10% or >25% categories include large numbers of species that often come from diverse realms, biomes or human biogeography regions. Consequently, the trends provided by these categories are ambiguous and hard to interpret. We therefore mostly focus on breakpoint and slope trends based on species that have >50% >75% >90% or >99% of their range within a specific region. These categories reflect increasing specificity of species towards a specific realm, biome or human biogeography region.

Given realm-specific breakpoints estimated when including species of increasing specificity, we observe a trend towards increasingly younger breakpoint estimates for the Afrotropic, Indomalayan and Nearctic realm, a slight trend towards older estimates for the Neotropic realm and no trend for the Palearctic (Supplementary Table 6 and Supplementary Fig. 3a). Therefore, breakpoint estimates usually show a trend towards younger estimates with increasing realm-specificity, but the clarity of this pattern is limited.

With increasing biome-specificity (Supplementary Table 7 and Supplementary Fig. 4a), breakpoint estimates for the tropical biome showed the clearest trend towards increasingly younger estimates and a trend towards older breakpoint estimates was observed for the arid biomes. When considered together with the >10% or >25% specificity categories, the temperate and polar biome showed a trend towards younger breakpoint estimates, while the cold biome showed a trend towards older breakpoint estimates. No general trends were observed across biomes, especially as species with >75%, >90% or >99% of their present natural ranges in the cold or polar biomes are not present in our dataset.

When considering increasing specificity for human biogeography regions, we observe the clearest trend towards younger breakpoint estimates for the region of late archaic arrival (Supplementary Table 8 and Supplementary Fig. 5a). Additionally, there is a trend towards younger breakpoint estimates for the *H. sapiens*-only region, but only when considering categories between >10% and >75% of species range within this region (the >90% and >99% categories are likely dominated by population dynamics of Australasian species where *H. sapiens* arrived relatively early compared to the Americas). Lastly, as observed in Supplementary Tables 1 and Supplementary Tables 4-5, slope estimates for the period after the breakpoint (Supplementary Fig. 3b, Supplementary Fig. 4b and Supplementary Fig. 5b) tended to have negative values. This negative relationship is likely an indicator of increased severity of population decline towards present time, as described in the decline severity analysis in the main text (Fig. 1c).

Supplementary Note 3: Phylogeny effect

To test the effect of phylogenetic relationships between megafauna species on the inferred patterns in Fig. 1, we first subset our dataset of 139 megafauna species such that we retained only a single representative species per genus, which left us with 67 species in total (Supplementary Fig. 8 and Supplementary Table 9). This allowed us to test whether population decline patterns remain similar when maximizing phylogenetic distance (and thus minimizing the probability of shared evolutionary history) between species. We conducted a piecewise linear regression on the estimated population size dynamics of the species subset and observed two periods of differing decline intensity - with the more ancient global slope (before 55,793±2,343 years) estimated to be -1.43×10^{-8}; 95% CI [-2.2×10^{-8}, -6.54×10^{-9}] and the more recent slope (after 5,793±2,343 years) estimate to be -2.1×10^{-5}; 95% CI [-2.35×10^{-5}, -1.85×10^{-5}] - in line with the full dataset analysis (Fig. 1 and Supplementary Table 1). The global breakpoint between the two periods of decline using the species subset was somewhat shifted towards the past (55,254±2,343 years ago), but remained very similar to the one estimated using the full dataset (Supplementary Table 1). We also investigated decline severity (Fig. 1c and Supplementary Note 1) of the species subset and again found similar patterns to the full dataset analysis, with 95% of the species experiencing extremely strong decline severity (between 78.4% and 99.9%).

We next conducted a phylogenetic regression analysis using the MCMCglmm R library[9]. Specifically, we used the phylogenetic trees (Supplementary Fig. 9) provided in the PHYLACINE database[4] to control for the phylogenetic relatedness between megafauna species and fitted a linear model with the median slope of decline as the response variable, the species' adult mass as the explanatory variable and the species phylogeny as the random effect. This dataset consisted of 100 species for which we found an equivalent in the PHYLACINE database (Supplementary Data 2). We ran three chains of the Markov Chain Monte Carlo (MCMC) regression algorithm for each of the 1,000 phylogenetic trees provided in the PHYLACINE database, and extracted regression coefficients for each iteration of the MCMC algorithm. The median regression coefficient for the decline~mass relationship was estimated to be -0.112 (95% HPDI: [-0.167, -0.067]) across MCMC iterations and thus very similar to the estimated coefficient of the full dataset analysis (95% HPDI: [-0.152, -0.059]), while the random phylogeny effect was estimated to be -0.015 (95% HPDI: [-0.125, 0.092]). The median estimate of Pagel's λ[10] - a quantification of the phylogenetic signal in trait evolution ranging from 0 to 1, with values closer to 0 indicating that closely related species are not more similar for the trait value compared to distant relatives - was estimated to be 0.336 (95% HPDI: [0.124, 0.590]) across MCMC iterations. Together, these results indicate a negligible influence of phylogenetic relationships on megafauna population dynamics.

Supplementary Note 4: Human-megafauna PSMC trajectory relationship

In order to infer the relationship between the effective population size trajectories of the human and megafauna populations we adopted the approach outlined in Chen et al. (2019)[11]. We first simulated a hybrid African-Asian human sequence using the cosi1.2 software under the assumption of the best-fitting model of human demography estimated by Schaffner et al. (2005)[12]. The combined human sequence is meant to mimic the global expansion of *Homo sapiens*, given that it contains a global representation of genetic variation accumulated in different human populations - in this case, the African and Asian sequences were chosen as they are the most geographically and genetically diverged populations considered by Schaffner et al. (2005). In total, we simulated five 30 megabase hybrid sequences which were then used as input for inference of the human population trajectory using the PSMC method[1] (Supplementary Fig. 14). Additionally, we have also inferred population size trajectories from empirical high-coverage data of an African and Asian individual available from the 1000 Genomes Project[13] - individuals NA19239 and NA18524, respectively.

We observe a correspondence of the three human PSMC trajectories between 1,500,000 and 150,000 years ago, as well as negative correlation with global megafauna dynamics during this time frame (Spearman's ϱ = -0.89, $p < 0.001$ for the African individual; Spearman's ϱ = -0.62, $p = 0.024$ for the Asian individual; Spearman's ϱ = -0.81, $p < 0.001$ for the simulated African-Asian individual). However, between 150,000 and 50,000 years ago both the African and Asian populations show signs of bottleneck dynamics, likely due to extensive structuring and migrations of the human population during this period[14]. Consequently, when correlating the full trajectories (between 1,500,000 years ago and the present) of megafauna and human individuals, only the trajectory inferred from the simulated African-Asian genome still correlates negatively with the global megafauna trajectory (Spearman's ϱ = -0.81, $p < 0.001$), while the correlation becomes non-significant for the trajectories of the African and Asian individual (Spearman's ϱ = 0.004, $p = 0.987$ for the African individual; Spearman's ϱ = -0.01, $p = 0.967$ for the Asian individual). However, the population size trajectories inferred from both the African and Asian genomes start increasing between approximately 50,000 years ago and present time, in stark opposition to the megafauna trend, which experienced their lowest population sizes during this time frame (Supplementary Fig. 14).

Supplementary Note 5: Alternative quantification of past megafauna census sizes

To provide an alternative quantification of census sizes (N_c) of megafauna for different time periods, compared to using the N_e/N_c ratio (Fig. 4 and Supplementary Note 1), we utilise the positive relationship between effective and current IUCN census estimates (Spearman's ϱ = 0.510, p < 0.001; Supplementary Fig. 16). Specifically, we fit a linear model for the dependence of IUCN N_c estimates on Holocene N_e estimates (representative of the species' current N_e)

$$y_i \sim N(\mu_i, \sigma)$$
$$\mu_i = a + bE_i$$
$$a \sim N(5, 1)$$
$$b \sim N(0, 1)$$
$$\sigma \sim exp(1),$$

assuming a normal distribution (N) for the IUCN N_c estimates (y) and the priors of the intercept (a) and slope (b) of the relationship, and an exponential (exp) prior for the model error σ. The predictor variable E signifies the Holocene N_e estimates. Furthermore, both N_c and N_e estimates were $\log_{10}$-transformed prior to model fitting.

In total, we fit the model four times, using four different subsets of species, that are based on four different filtering procedures. The criteria that ensure inclusion of the species in the modelling step should reflect a meaningful, consequential relationship between IUCN N_c estimates and Holocene N_e estimates. This data filtering step is necessary, as the reductions of census sizes in some species are likely so severe that the relationship between N_c and N_e estimates is effectively decoupled. Additionally, the four different sets of estimates allow us to obtain broader ranges of parameters of interest. The inclusion criteria are as follows:

- species that have a higher census size compared to their Holocene effective size (N_c>N_e model; 83 species in total)
- species with a current geographic range that covers the entire present natural range as predicted by PHYLACINE[4] (*Range* model; 30 species in total)
- species of least concern (LC) according to the IUCN red list assessment (*LC* model; 29 species in total)
- species with a stable population trend according to the IUCN red list assessment (*Stable* model; 21 species in total)

By randomly sampling the inferred posterior distributions of intercepts (a) and slopes (b) of the models, we calculated posterior sample distributions for the total sum of N_c values across the 139 species in our dataset for the four time windows during the last 100,000 years, as well as the baseline time window corresponding to the period between 100-742 kya. Specifically, to obtain a single posterior sample of an N_c sum, we randomly sampled 139 intercept and slope values from the posterior distribution of the fitted model, used these values to predict N_c values for each of the 139 species (based on the corresponding N_e value of a specific time period), and lastly, summed across the 139 species- and time-specific N_c values. This procedure was repeated 1,000 times to obtain a posterior sample distribution for the N_c sum for each time interval of interest. Since we ran four different models, we obtained a posterior sample distribution containing 4,000 N_c values for each time window.

Posterior sample N_c distributions for the current period were generated in a similar way - by sampling posterior distributions of a and b parameters, generating N_c values, and then multiplying these value by a scaling factor

$$f = \frac{\sum_i N_c}{\sum_i N_c^{EST}},$$

where $\sum_i N_c$ is the sum of IUCN census sizes across all species (including species with $N_e > N_c$) for which this estimate is available, while $\sum_i N_e^{EST}$ is the sum of the medians of the posterior distributions of census sizes across the corresponding species, estimated for the Holocene period. The four different scaling factors for the four different models were

$$f(N_c > N_e) = 0.825754$$
$$f(Range) = 0.413487$$
$$f(LC) = 0.4133966$$
$$f(Stable) = 0.359657,$$

reflecting the difference in current IUCN census sizes and model-predicted census sizes for the Holocene. The difference between the two sums used to calculate f comes from the fact that the predictive models are based on subsets of species that conform to the filtering criteria and thus, less severely bottlenecked species. Consequently, the posterior prediction of Holocene N_c is higher than the currently observed IUCN values. In effect, the model predicts the Holocene census sizes that would be expected if severe bottlenecks did not occur. Therefore, to obtain more realistic N_c values for the current period, f serves as a correction factor, as it reflects the average reduction between model-predicted N_c values, that are

estimated under the assumption of lower bottleneck severities, and the observed IUCN N_c dataset, that includes estimates of severely bottlenecked species.

Posterior sample distributions for total biomass and energy turnover were estimated in the same way as the posterior sample distributions of N_c sums, but prior to summing across the 139 species- and time-specific N_c values, the N_c values were multiplied by the corresponding adult mass of each species (as reported by PHYLACINE) or daily metabolic rate from Pedersen *et al.* (2023)[5].

In order to obtain N_c values, biomass and energy turnover for species missing in our dataset (i.e., extant megafauna species for which suitable genomic data were unavailable, as well as extinct megafauna species), we first used the PHYLACINE database to extract species information of all extant and extinct terrestrial mammals above or equal to 22 kg (corresponding to the mass of the smallest megafauna species included in our original dataset). We then filtered out species that were included in the original 139-species dataset, which left us with 318 missing megafauna species (121 extant and 197 extinct megafauna species). For each of these species we extracted adult mass data using PHYLACINE and daily metabolic rates from Pedersen *et al.* (2020)[15].

Using the original 139-species dataset we then fitted the linear model

$$y_i \sim N(\mu_i, \sigma)$$
$$\mu_i = a + bm_i$$
$$a \sim N(5, 1)$$
$$b \sim N(0, 1)$$
$$\sigma \sim exp(1),$$

where y is the N_c value of a species and m is the adult mass. We assumed a normal distribution (N) for the N_c values (y) and the priors of the intercept (a) and slope (b) of the relationship, and an exponential (exp) prior for the model error σ. Both N_c and m were $\log_{10}$-transformed prior to model fitting. The y response variable consisted of all N_c values within the posterior sample distributions across all species, estimated by the previous model. We fitted this model to the N_c posterior sample distribution estimated for the baseline and current periods. As before, each of these two models was fitted four times, using the four different N_c posterior sample distributions corresponding to the four different subsets of species that are based on the different filtering procedures. We then used the fitted models and the mass values of missing megafauna species to predict their N_c posterior sample distributions for the baseline and current periods. As before, we used the four fitted models to obtain a 4,000-sample posterior distribution of N_c for each missing species and each time period. Posterior sample distributions for total biomass and energy turnover were estimated in the same way as for the previous model.

We calculated the median of the posterior sample distributions for each of these parameters for different time points and groups of species. We then expressed these values relative to the values of the baseline period (100-742 kya), resulting in a comparable representation of megafauna parameters to the one presented in Fig. 4 (Supplementary Fig. 17).

We calculated the medians of the posterior sample distributions across the four fitted models (based on the four different subsets of species and filtering criteria) and report the range of these medians for the original 139 species included in our dataset. We observed a continuous decline in total biomass starting between 75-100 kya, while total census size and energy turnover decreased below the baseline between 50-75 kya (Supplementary Fig. 17a and Supplementary Fig. 18a). At current time, the percent

change from the baseline reached a median reduction of ~75% (median range: 72-80%), ~77% (median range: 74-81%) and ~76% (median range: 73-80%) for census size, biomass and energy turnover, respectively. On average, this reduction equated to a total loss of ~115 million megafauna individuals, ~0.005 gigatonnes of carbon (Gt C) of biomass and a ~2.1 pJ/day of energy turnover over the last 100,000 years.

When including extinct species, as well as extant megafauna species that are missing from our dataset, we estimated that the global megafauna census was ~600 million individuals (range: 500-770 million individuals) across a total of 457 megafauna species (139 extant species comprising our initial study dataset, 121 extant species missing from this dataset and 197 extinct species) during the baseline period, which reduced by ~86%, i.e., to ~81 million individuals (range: 76-86 million individuals) during the current period. Similarly, total baseline biomass (~0.034 Gt C; range: 0.025-0.043 Gt C) and energy turnover (~10.88 pJ/day; range: 8.22-13.58 pJ/day), reduced by ~94% (currently, ~0.0021 Gt C; range: 0.0018-0.0023 Gt C) and ~90% (currently, ~0.99 pJ/day; range: 0.90-1.08 pJ/day), respectively (Supplementary Fig. 17b and Supplementary Fig. 18b). Furthermore, the proportion of total megafauna census, biomass and energy turnover contributed by extinct species during the baseline period was estimated to be ~44%, ~73%, and ~60%, respectively.

To gauge the extent of the potential underestimation of megafauna parameters, we made a comparison to the historical census size estimates of megafauna. The 19th century estimate of bison population size (30 million)[6] is within the upper 99th percentile of our baseline N_c estimate for this species (Supplementary Fig. 19a), while the estimate of 27 million individuals for elephants[7] exceeds their highest estimated baseline N_c by ~14% (Supplementary Fig. 19b). If we consider the estimates over the 99th percentile of the total sum of census sizes across megafauna species, this would equate to an estimate of >15 billion megafauna individuals, >1 Gt C of biomass and >400 pJ/day of energy turnover during the baseline period (Supplementary Table 12).

The estimated parameter distributions vary over orders of magnitude (Supplementary Fig. 18) and thus likely capture the majority of the actual range for the parameters. Albeit, the large variance of these distributions is likely a consequence of the moderate correlation strength between the two population size measures (Supplementary Fig. 16). Additionally, the median reductions in megafauna parameters between the baseline and current periods are somewhat smaller (Supplementary Fig. 17) compared to the estimates presented in the main text (Fig. 4). This could be a consequence of the measures of the population size being $\log_{10}$-transformed, and thus a compression of the variance, prior to model fitting, whereas the main text analysis uses the N_e/N_c ratio based on untransformed measures. Consequently, the analysis based on $\log_{10}$-transformed values, likely represents a lower bound of the consequences of megafauna decline. Importantly however, while both analyses produced relatively similar estimates of the relationship between baseline and current megafauna parameters, they are likely to underestimate consequences of megafauna decline as they are both based on current N_c values of species that underwent population contractions in the recent past.

Supplementary Note 6: Comparison of interglacial population dynamics

The Last Interglacial (Marine Isotope Stage 5e; ~116-129 thousand years ago) preceded widespread megafauna decline[16]. It is also the most recent point at which, under conditions similar to today, historic population sizes were still intact (Fig. 2a). The Last Interglacial was 0.5 ± 0.3°C warmer[17] than pre-industrial global temperatures and, whilst not an exact climate analogue, largely characterised by climates comparable to the present[18]. The proximity of the Last Interglacial to today means that the two time periods are evolutionarily similar, and finer-resolution data is available compared to previous periods. As a result, research has considered the Last Interglacial as an important baseline for biodiversity restoration, as well as a testbed for future climate projections. Here, the Last Interglacial presents an appropriate baseline with which to compare the reduced megafauna populations of the present.

We compared megafauna census sizes, biomass and energy turnover between the Last Interglacial and the current period. The methods for estimating these parameters correspond to the inference presented in the main text (Fig. 4a), as described in the Methods section and Supplementary Note 1. Similarly to the main text results, we observed that the percent change from the Last Interglacial to current time reached a reduction of ~98%, ~94% and ~93% for census size, biomass and energy turnover, respectively (Supplementary Fig. 20). The absolute loss of individuals, biomass and energy turnover were estimated to be ~1.4 billion, ~0.014 Gt C and ~5.6 pJ/day, respectively.

We have also estimated the lower bound of these parameters following the description in Supplementary Note 5 and observed that the percent change from the Last Interglacial to current time reached a median reduction of at least ~77% (median range: 72-80%), ~76% (median range: 74-81%) and ~76% (median range: 73-80%) for census size, biomass and energy turnover, respectively (Supplementary Fig. 21a). The absolute loss of individuals, biomass and energy turnover were estimated to be ~122 million, ~0.0048 Gt C and ~2.1 pJ/day, respectively (Supplementary Fig. 21b).

We next conducted separate parameter estimation for each biogeographic realm to characterise the variance in the lower bound of megafauna loss across different geographical regions. These results show that all realms have been severely affected. Notably, while the Afrotropic realm is renowned for having suffered fewer megafauna extinctions during the late Quaternary compared to other realms and still harbors a rich megafauna, its surviving large-bodied species nevertheless suffered major declines. We observed the smallest, but still high, percent loss of megafauna individuals for the Afrotropic realm (~72%) and the largest for Australasia (~87%), with the rest of the realms in the range of ~77-81% loss (Supplementary Fig. 22a). Similar trends of reduction were observed for biomass (~75% for Afrotropics and ~89% for Australasia; ~77-80% for the rest of the realms) and energy turnover (~73% for Afrotropics and ~87% for Australasia; ~77-81% for the rest of the realms). In absolute terms (Supplementary Fig. 22b), the largest loss of individuals per species occurred in the Palearctic (~860,000 individuals per species) and the Neotropic realm (~800,000 individuals per species), while the smallest loss occurred in Indomalaya (~560,000 individuals per species) and the Nearctic (~620,000 individuals per species). On the other hand, the largest loss of biomass per species occurred in the Afrotropic realm (~4.5×10^{-5} Gt C per species) and Indomalaya (~4.05×10^{-5} Gt C per species), while the smallest loss occurred in Australasia (~4.5×10^{-6} Gt C per species) and the Nearctic (~9×10^{-5} Gt C per species). Lastly, the largest loss of energy turnover per species occurred in the Afrotropic (~1.7×10^{-5} pJ/day per species) and Palearctic realm (~1.5×10^{-5} pJ/day per species), while the smallest loss occurred in Australasia (~3×10^{-6} pJ/day per species) and the Nearctic (~6×10^{-6} pJ/day per species).

Supplementary Figures

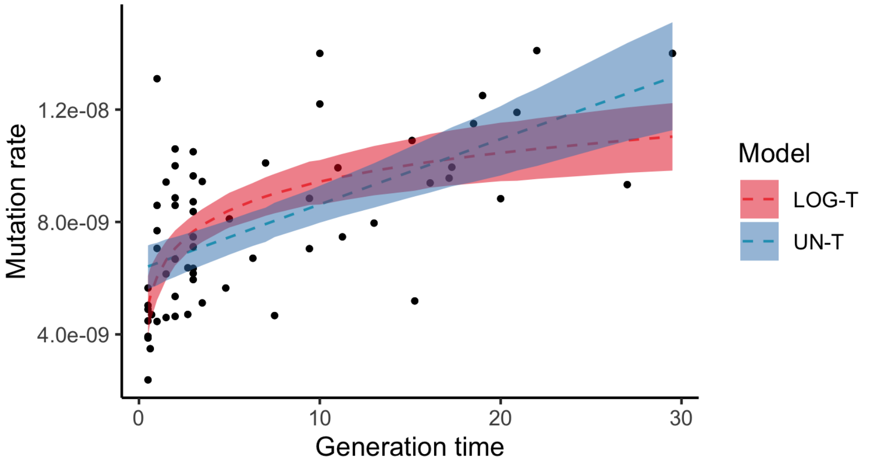

Supplementary Figure 1. Linear regression model using mammalian trio data from Bergeron et al. (2022) with per generation mutation rate as the response variable. The points are the observed data and two different fits depict linear models where the predictor (generation time) was log-transformed (LOG-T) or untransformed (UN-T), respectively. The dashed lines represent the fits of the models and the shaded areas are 95% confidence intervals for each model.

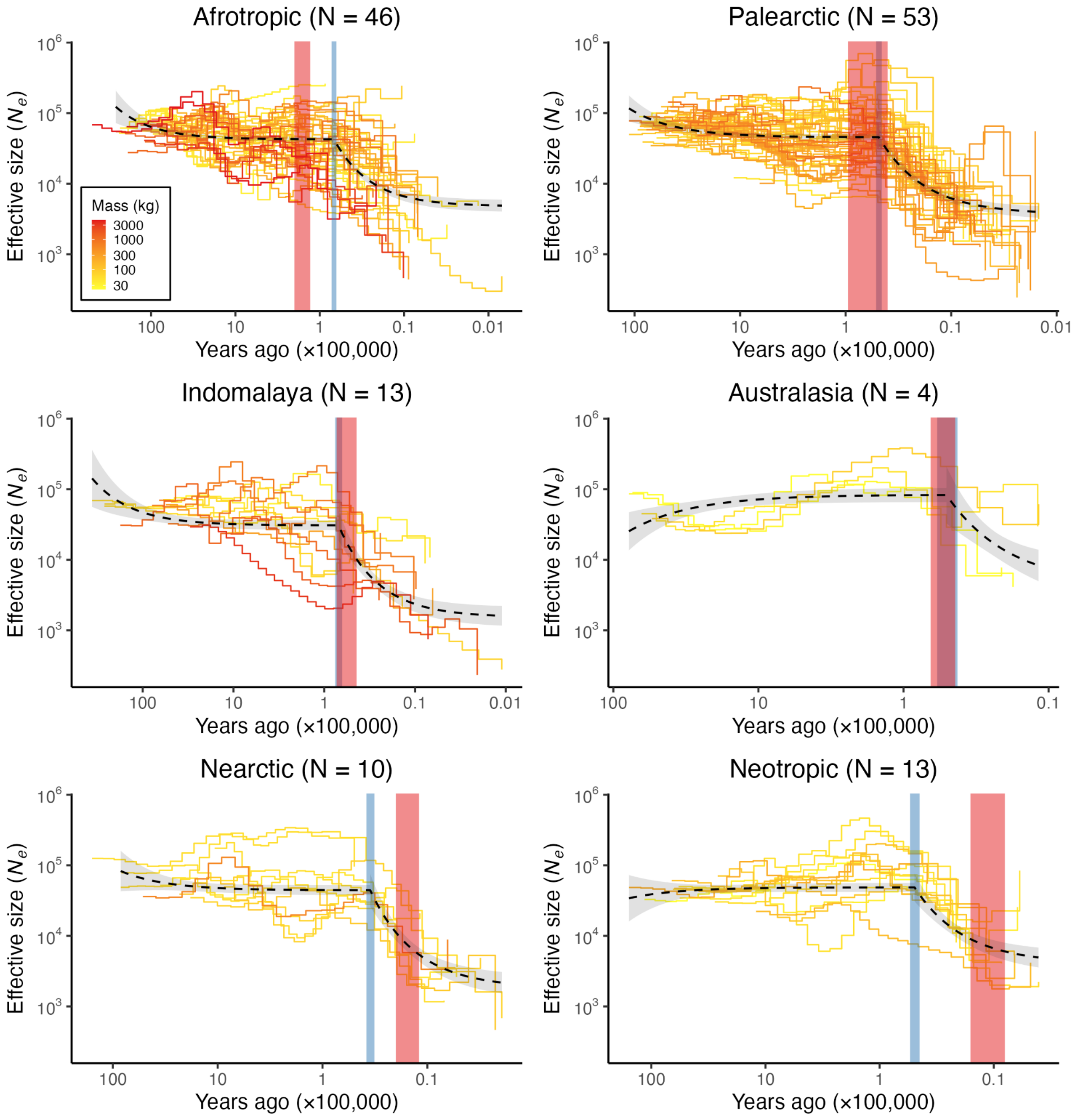

Supplementary Figure 2. Effective population size (N_e) dynamics of 139 extant megafauna species separated by biogeographic realm. The number of species in each realm is presented in the parentheses. The dashed lines represent the realm-specific fits of the piecewise linear model, as determined by breakpoint analysis (Supplementary Table 1). The blue rectangles represent the timespan of realm-specific breakpoints, while the red rectangles represent *H. sapiens* arrival ranges to each region or, in the case of Afrotropic realm, the timeframe of expansion of *H. sapiens* throughout the Afrotropical realm (Supplementary Table 3). The dashed line represents the fit of the piecewise linear model, as determined by breakpoint analysis. The grey-shaded area represents the 95% confidence interval of the linear model prediction. All axes are $\log_{10}$-transformed.

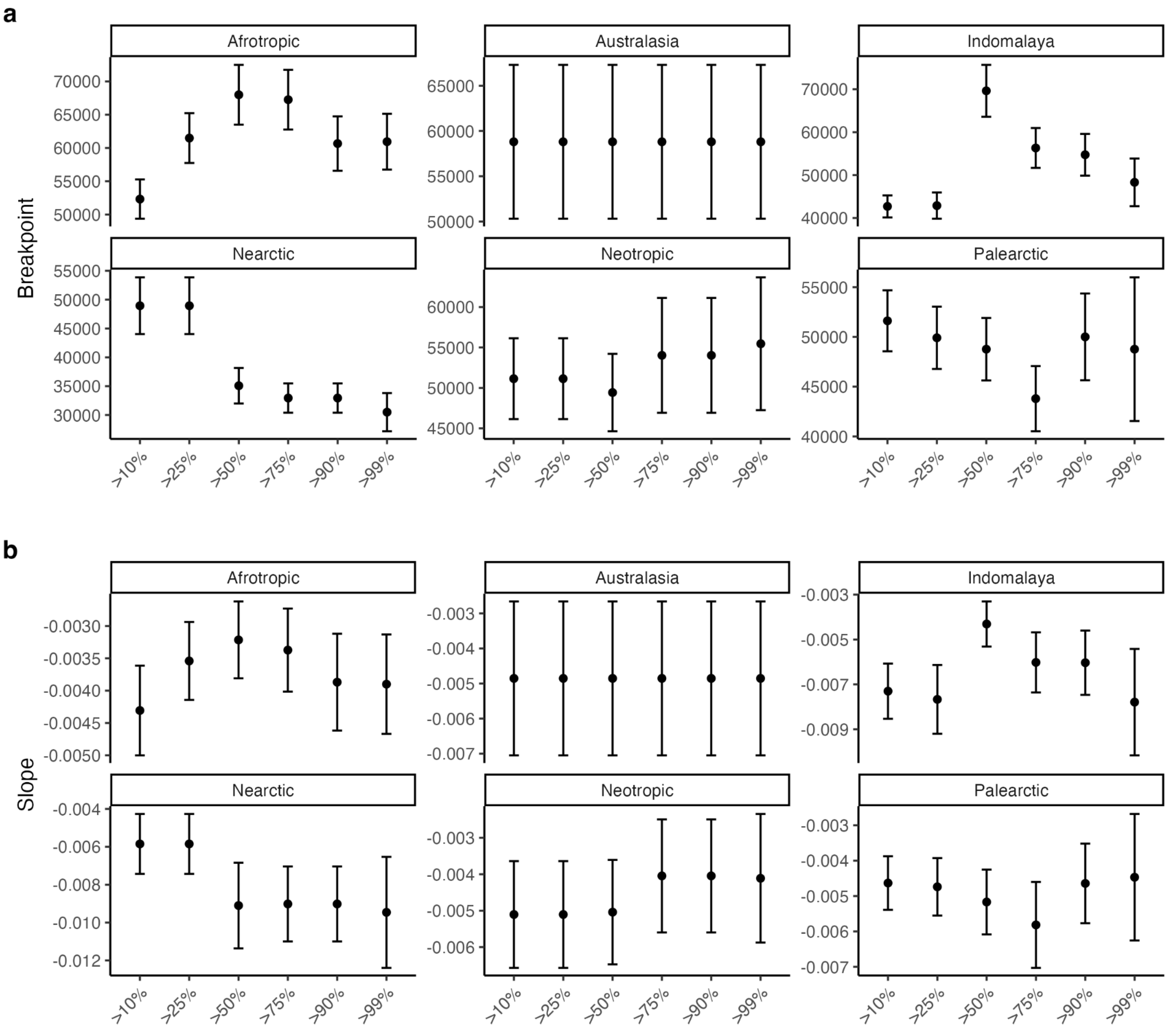

Supplementary Figure 3. **a** Breakpoint estimates and **b** slope estimates for the period after the breakpoint with respect to different percentage levels of species' ranges contained within a biogeographic realm. Points are mean breakpoint (or slope) values with 95% confidence intervals indicated by bars.

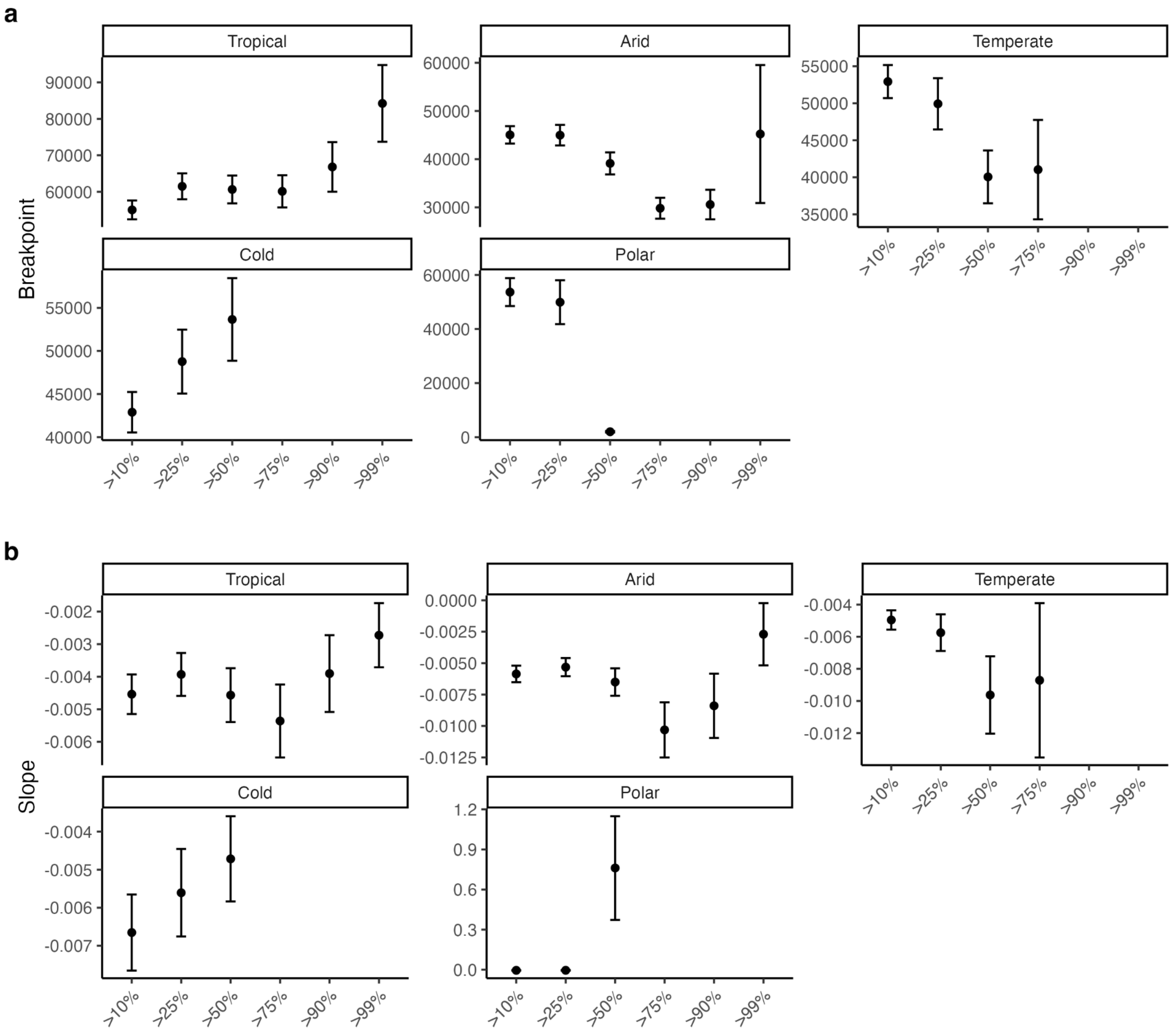

Supplementary Figure 4. a Breakpoint estimates and **b** slope estimates for the period after the breakpoint with respect to different percentage levels of species' ranges contained within a biome. Points are mean breakpoint (or slope) values with 95% confidence intervals indicated by bars.

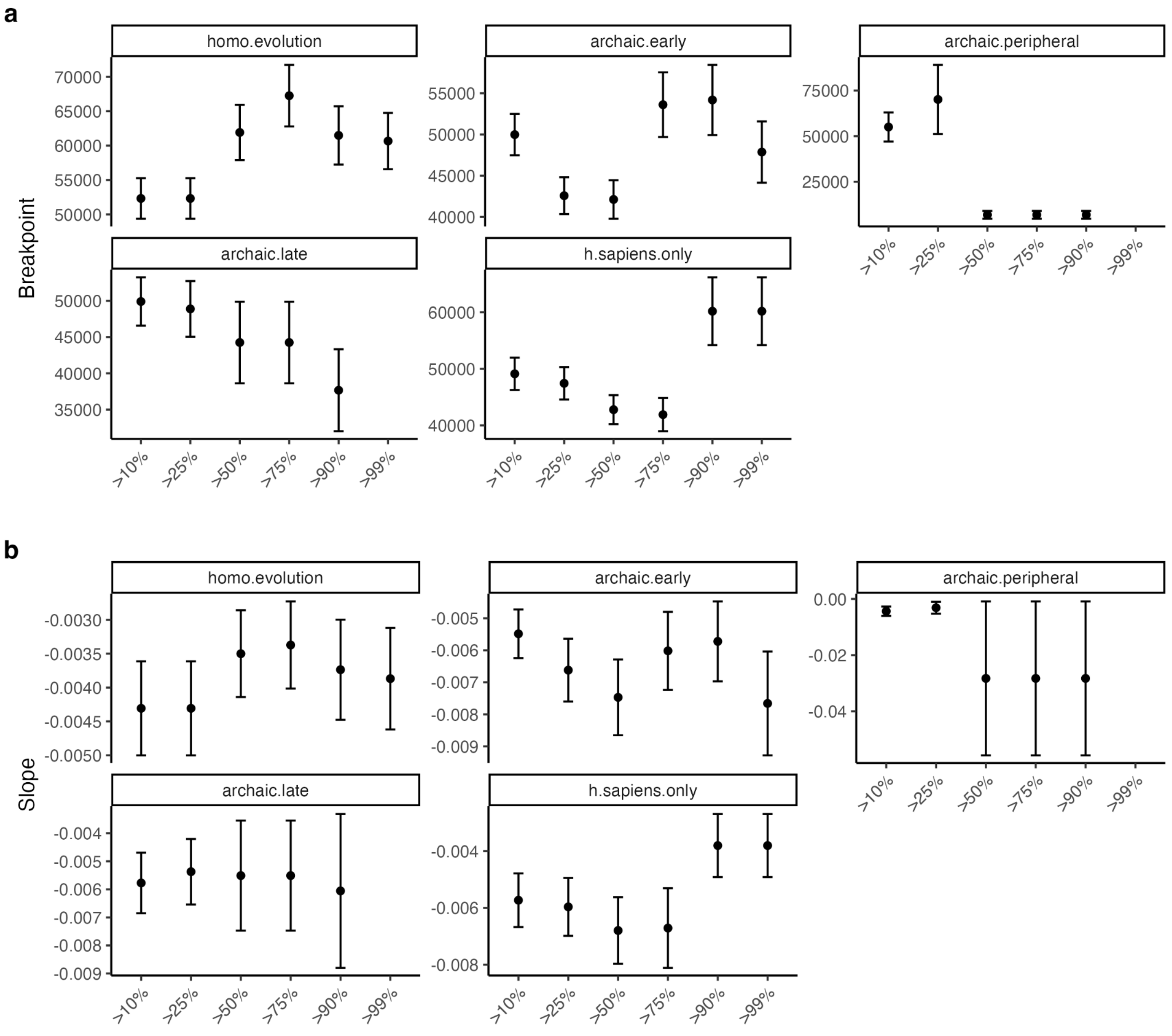

Supplementary Figure 5. a Breakpoint estimates and **b** slope estimates for the period after the breakpoint with respect to different percentage levels of species' ranges contained within a human biogeography region. Points are mean breakpoint (or slope) values with 95% confidence intervals indicated by bars.

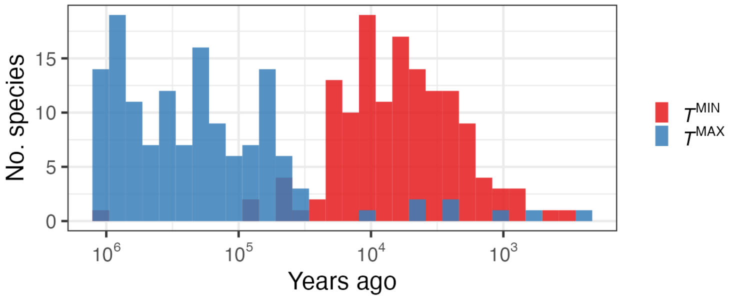

Supplementary Figure 6. Distributions of times since species experienced their highest population size (T^{MAX}) and lowest population size (T^{MIN}).

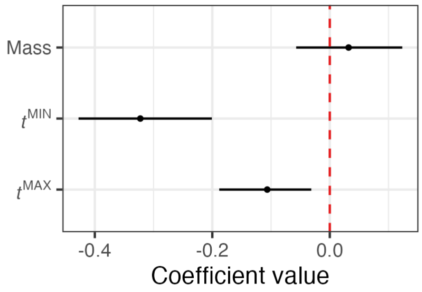

Supplementary Figure 7. Coefficient values of explanatory variables (species' adult mass; T^{MIN}: time when a species achieved the lowest population size, T^{MAX}: time when a species achieved the highest population size) for a regression model with species' decline severity as the response variable (Supplementary Note 1). The distribution for each coefficient is the 95% HPDI, with the point representing the median.

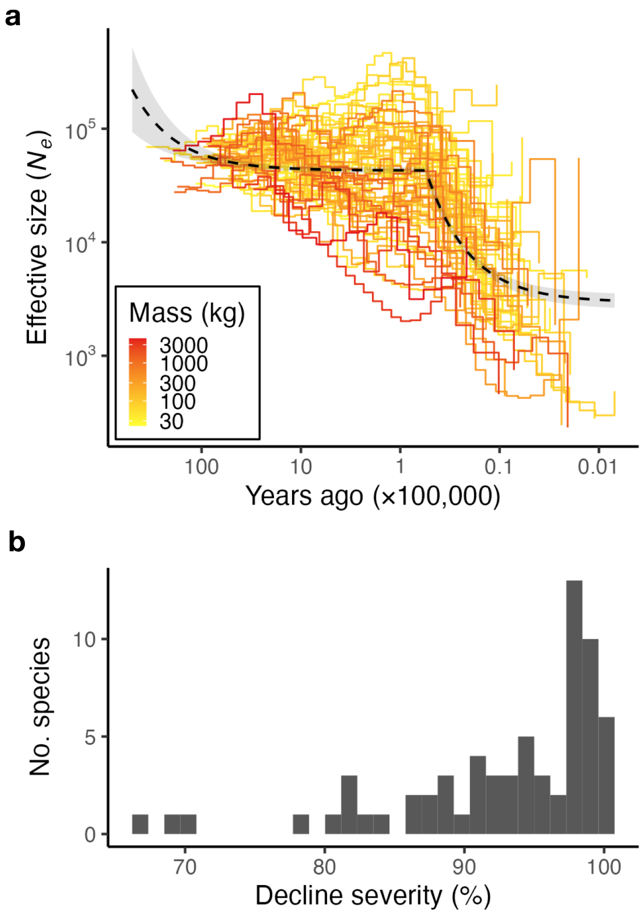

Supplementary Figure 8. Effective population size (N_e) trajectories of 67 extant megafauna species (one representative species per genus; Supplementary Table 9). a Each step line represents changes in N_e with respect to time for a single megafauna species, colored by a gradient based on average adult mass. The dashed line represents the fit of the piecewise linear model, as determined by breakpoint analysis. The grey-shaded area represents the 95% confidence interval of the linear model prediction. Both axes are $\log_{10}$-transformed. **b** Distribution of species' decline severity.

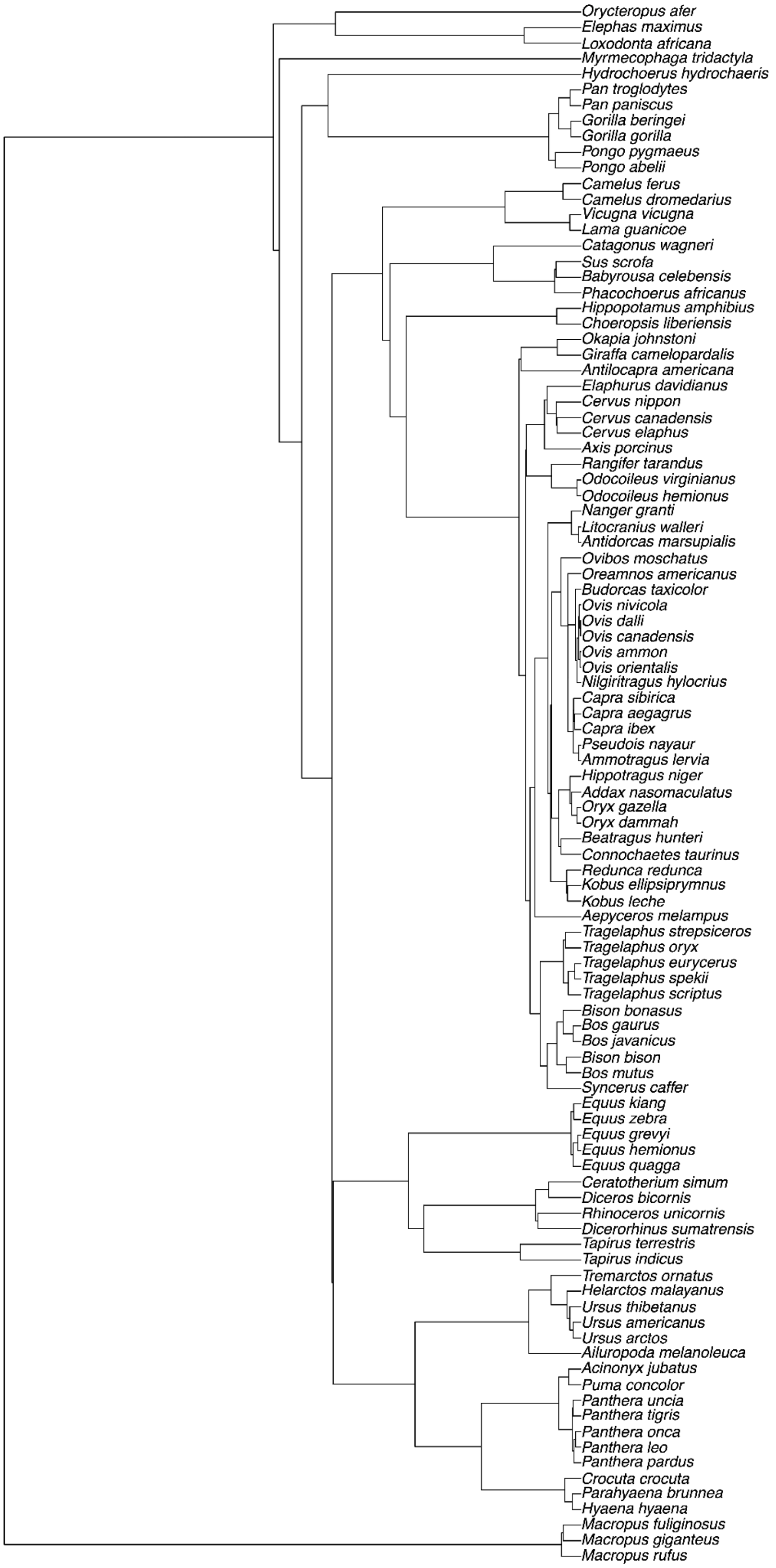

Supplementary Figure 9. One iteration of the phylogenetic tree from the PHYLACINE database used in the phylogenetic regression analysis.

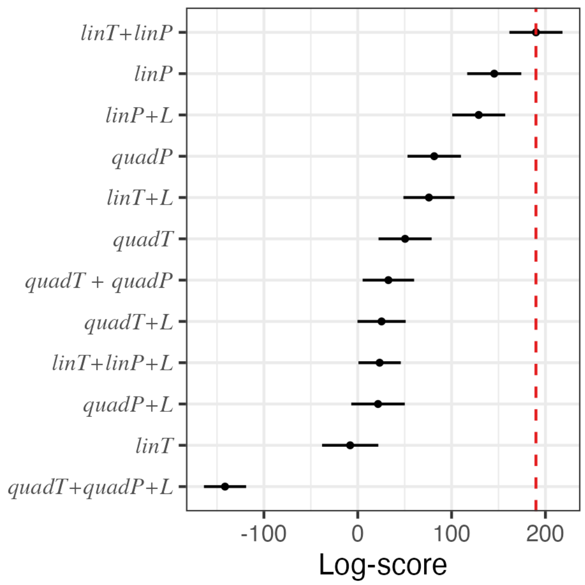

Supplementary Figure 10. Log-scores of leave-one-out cross-validation for 12 climate-based fitted models (Supplementary Table 10), with the red dashed line indicating the best-fitting model. *lin* and *quad* indicate models with a linear and quadratic effect of temperature (*T*) and/or precipitation (*P*) on population trajectories, respectively; *L* indicates the inclusion of the temperature and/or precipitation lag predictor in the model (Supplementary Note 1). Points are mean log-scores values with ±1 standard error indicated by bars (n = 1,000).

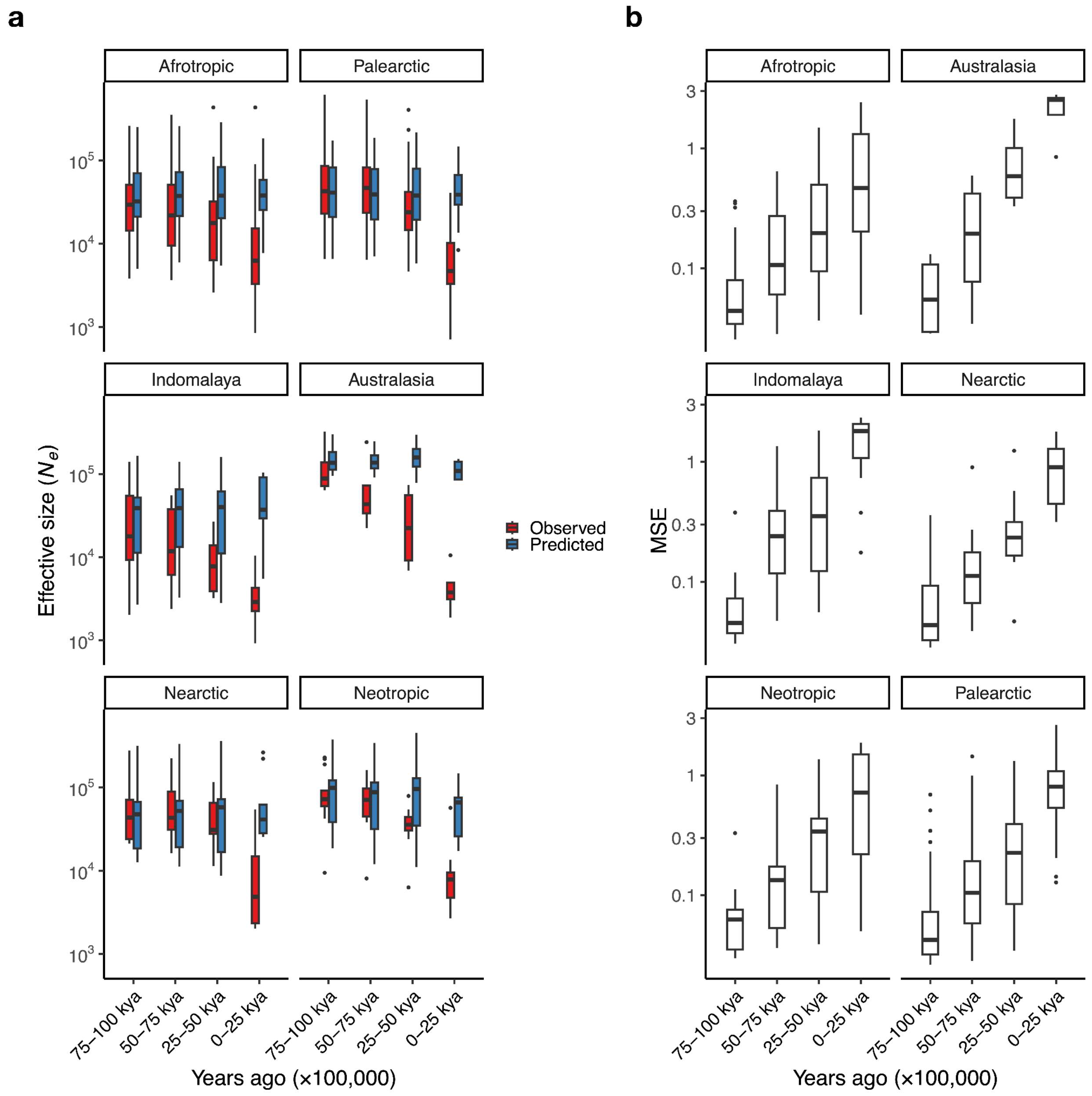

Supplementary Figure 11. a Realm-specific distributions of observed and predicted mean population sizes across species for four time intervals during the last 100,000 years, estimated using the best-fitting climate-based model. **b** The corresponding mean squared difference between observed and predicted population sizes (MSE). Each boxplot contains n = 139 species-specific N_e values. The median of the distributions is represented by the horizontal line within the boxes and box edges represent the interquartile range (IQR; 25th to 75th percentile). The upper whisker extends from the upper box edge to the largest value no further than 1.5×IQR and the lower whisker extends from the lower box edge to the smallest value at most 1.5×IQR. Values outside whiskers are represented as individual points. All y-axes are $\log_{10}$-transformed.

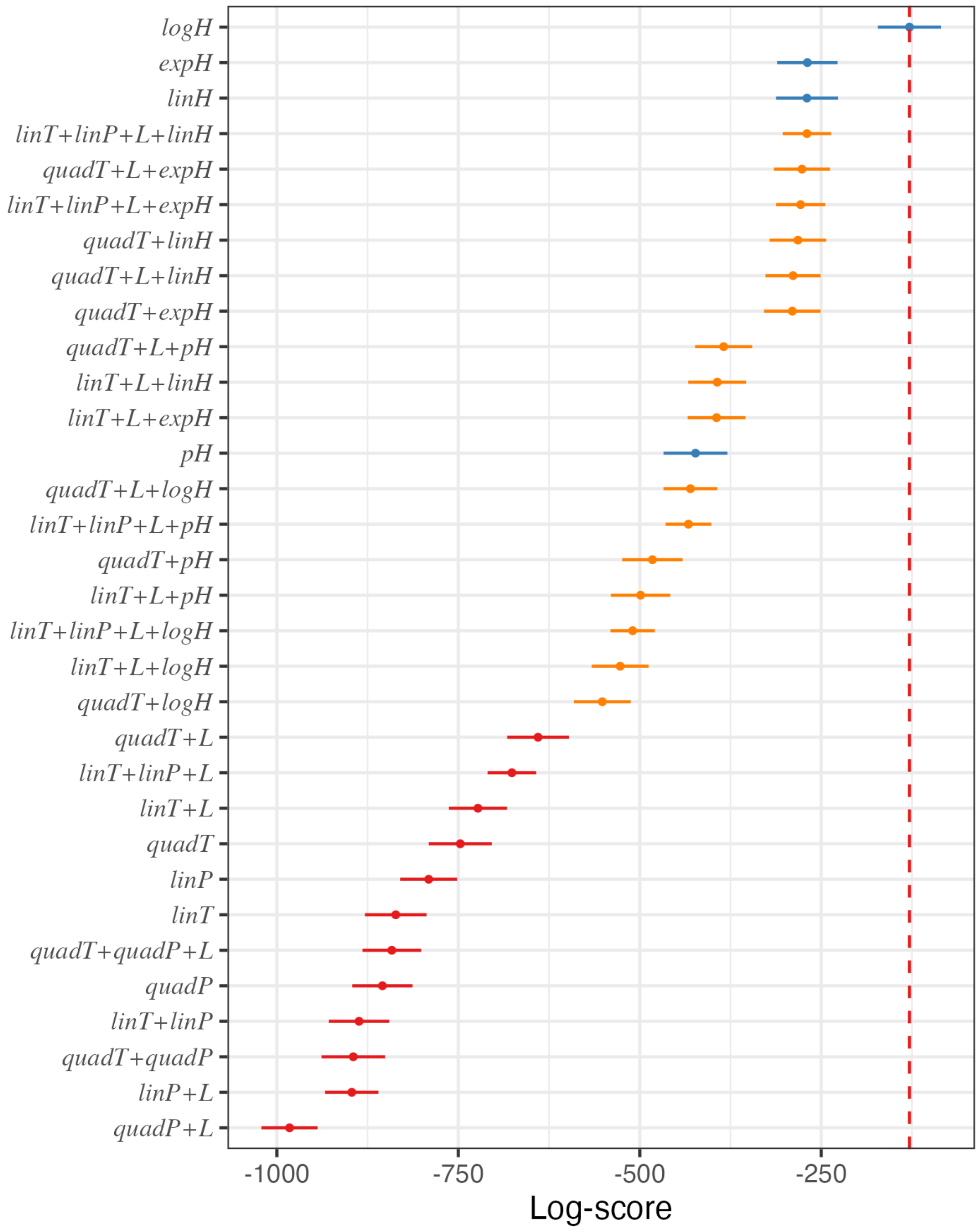

Supplementary Figure 12. Log-scores of leave-one-out cross-validation for all 32 fitted models (Supplementary Table 10), with the red dashed line indicating the best-fitting model. The colours red, blue and orange signify climate-only, human-only and combined models, respectively. Points are mean log-scores values with ±1 standard error indicated by bars (n = 1,000).

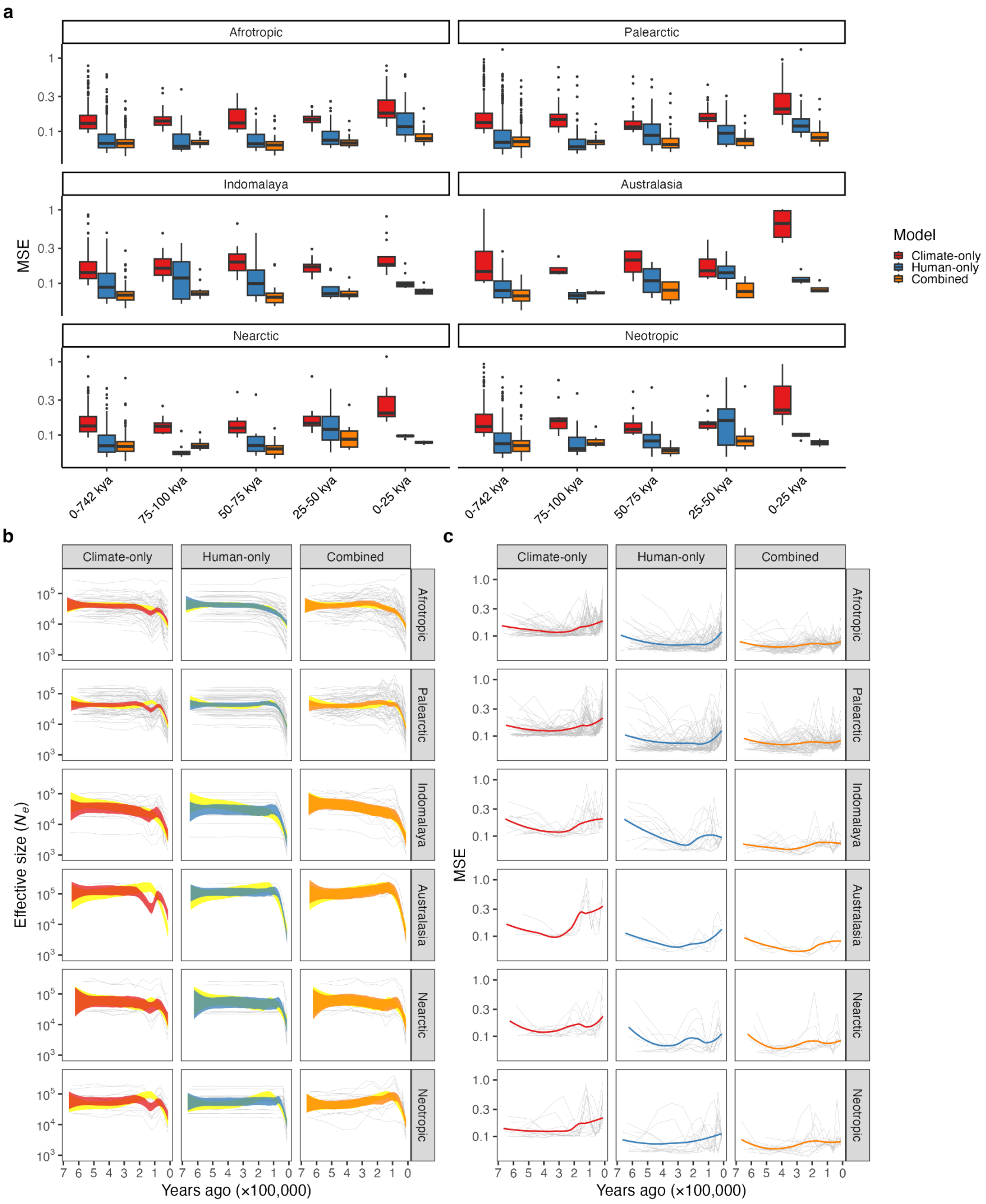

Supplementary Figure 13. a Realm-specific distributions of mean squared difference between observed and predicted population sizes (MSE) across species for the best-fitting model in each model class, for the whole time span (0-742 kya) and four time intervals during the last 100,000 years. For the whole time span (0-742 kya), each box-plot contains n = 1,470 species-specific N_e values, while n = 139 species-specific N_e values in each box-plot of the 25,000-year time intervals. The median of the distributions is represented by the horizontal line within the boxes and box edges represent the interquartile range (IQR; 25th to 75th percentile). The upper whisker extends from the upper box edge to the largest value no further than 1.5×IQR

and the lower whisker extends from the lower box edge to the smallest value at most 1.5×IQR. Values outside whiskers are represented as individual points. The y-axis is log_{10}-transformed. **b** The observed and predicted population size trends of megafauna, given the best-fitting model in each model class, separated by biogeographic realm. The yellow area is the mean observed population size trend of the realm, while each grey line represents the median predicted trend for a single species. The red, blue and green areas are the mean predicted population size trends across species for the best-fitting climate-only, human-only and combined model, respectively. The y-axis is log_{10}-transformed. **c** The corresponding MSE values. The y-axis is log_{10}-transformed.

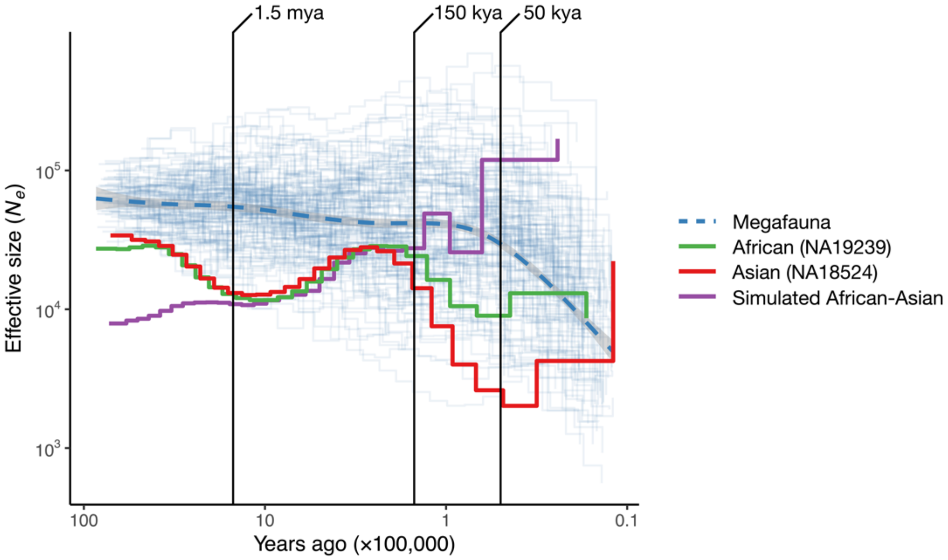

Supplementary Figure 14. Effective population size (N_e) trajectories of the human populations and 139 extant megafauna species (blue lines). The blue dashed line is the average population size trend across all species and gray ranges represent the 95% confidence interval, calculated using loess regression.. Both axes are log_{10}-transformed.

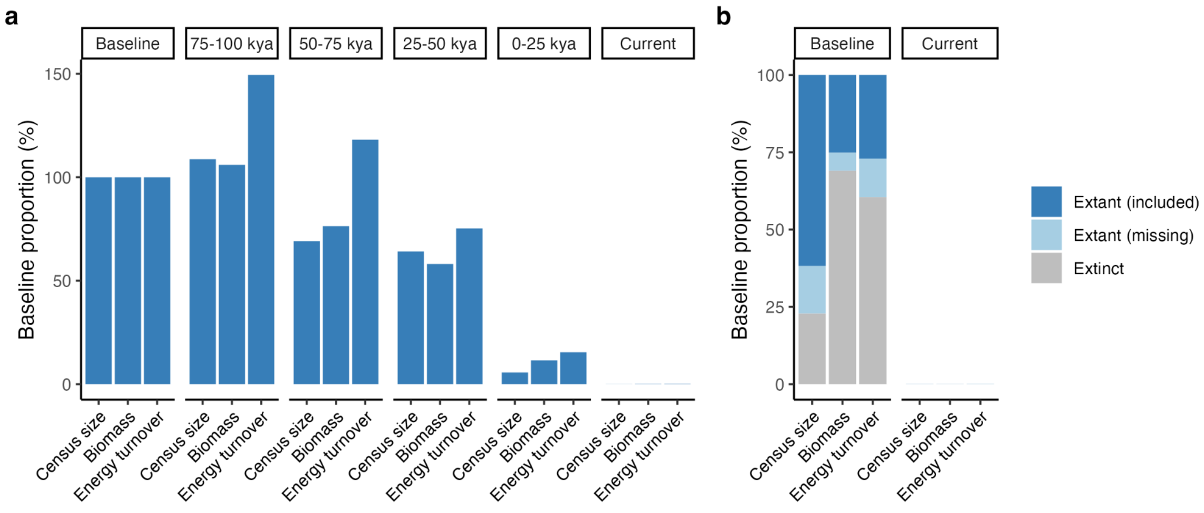

Supplementary Figure 15. Estimates of total megafauna individuals (Census size), biomass and energy turnover summed across species for different timepoints with respect to the baseline period using the r_{mod} ratio (Supplementary Note 1). a Parameter trends during the last 100,000 years with respect to the baseline period (100-742 kya) for 139 extant megafauna species that are included in our initial study dataset. **b** Contrast between the baseline and current period while taking into account all extant (both included or missing from our dataset) and extinct megafauna species (457 species for the baseline period and 260 species for the current period).

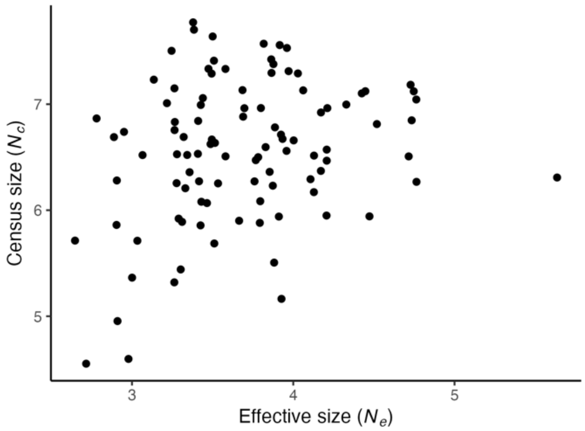

Supplementary Figure 16. Relationship between Holocene effective size (N_e) calculated as the average for the period between 0 to 11,700 years ago and current census size (N_c) for 99 megafauna species with available IUCN N_c estimates. Both axes are log_{10}-transformed.

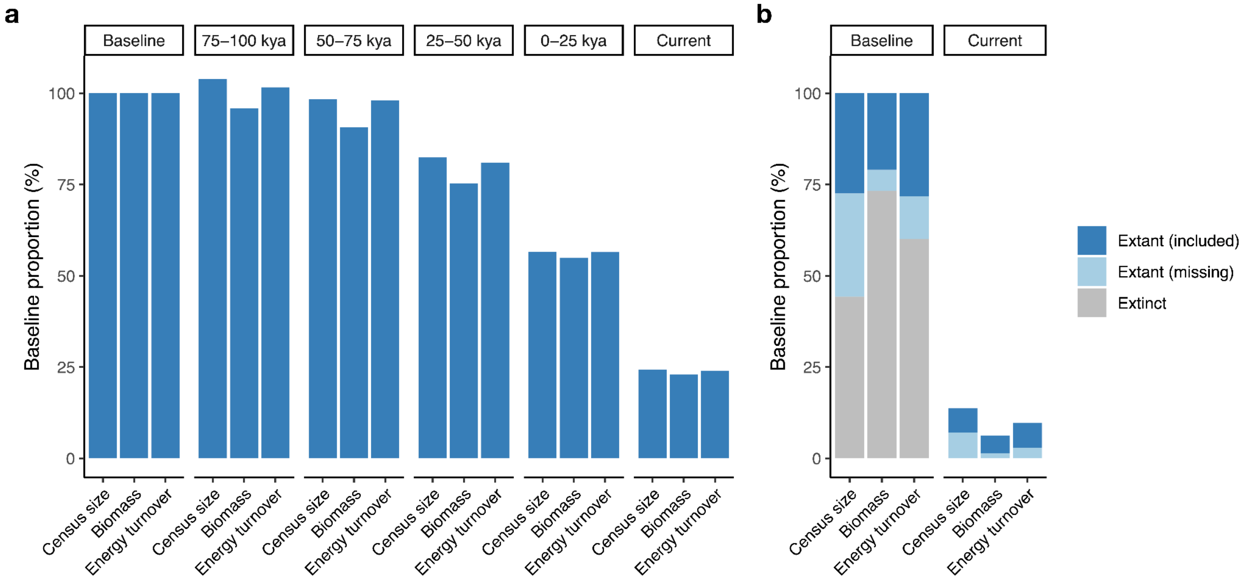

Supplementary Figure 17. Estimates of total megafauna individuals (Census size), biomass and energy turnover summed across species for different timepoints with respect to the baseline period using approach described in Supplementary Note 5. a Parameter trends during the last 100,000 years with respect to the baseline period (100-742 kya) for 139 extant megafauna species that are included in our initial study dataset. **b** Contrast between the baseline and current period while taking into account all extant (both included or missing from our dataset) and extinct megafauna species (457 species for the baseline period and 260 species for the current period).

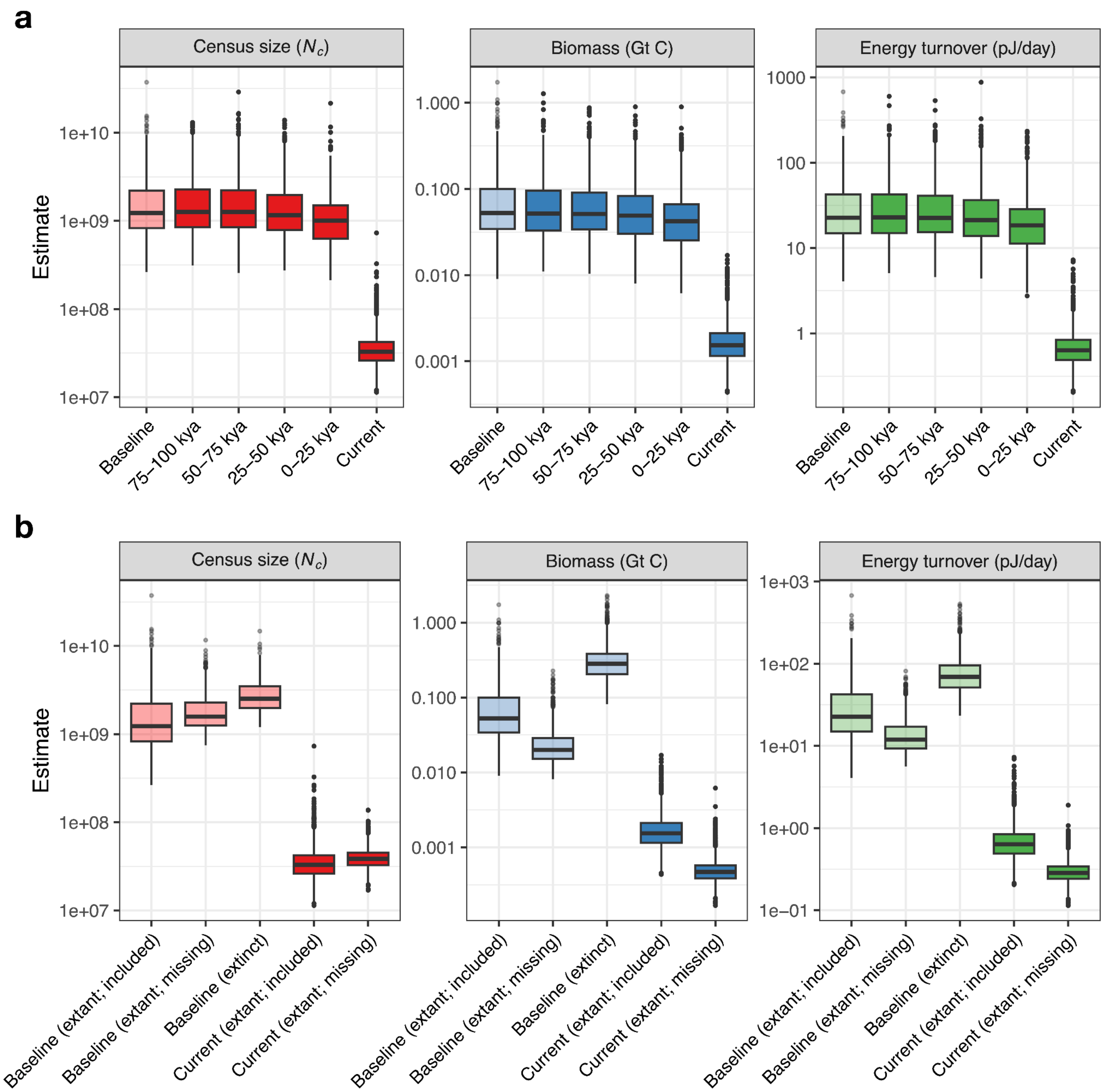

Supplementary Figure 18. a Posterior sample distributions for the total number of megafauna individuals (Census size), biomass (measured in gigatonnes of carbon; Gt C) and energy turnover (measured in petajoules per day; pJ/day) summed across species for time periods during the last 100,000 years and the baseline period (100-742 kya). Only the 139 extant megafauna species that are included in the original dataset are shown. **b** Posterior sample distributions of parameters for extant (both included in the original dataset and missing species) and extinct species during the baseline and current periods. All distributions consist of n = 4,000 posterior samples (Supplementary Note 1). The median of the distributions is represented by the horizontal line within the boxes and box edges represent the interquartile range (IQR; 25th to 75th percentile). The upper whisker extends from the upper box edge to the largest value no further than 1.5×IQR and the lower whisker extends from the lower box edge to the smallest value at most 1.5×IQR. Values outside whiskers are represented as individual points. All y-axes are log_{10}-transformed.

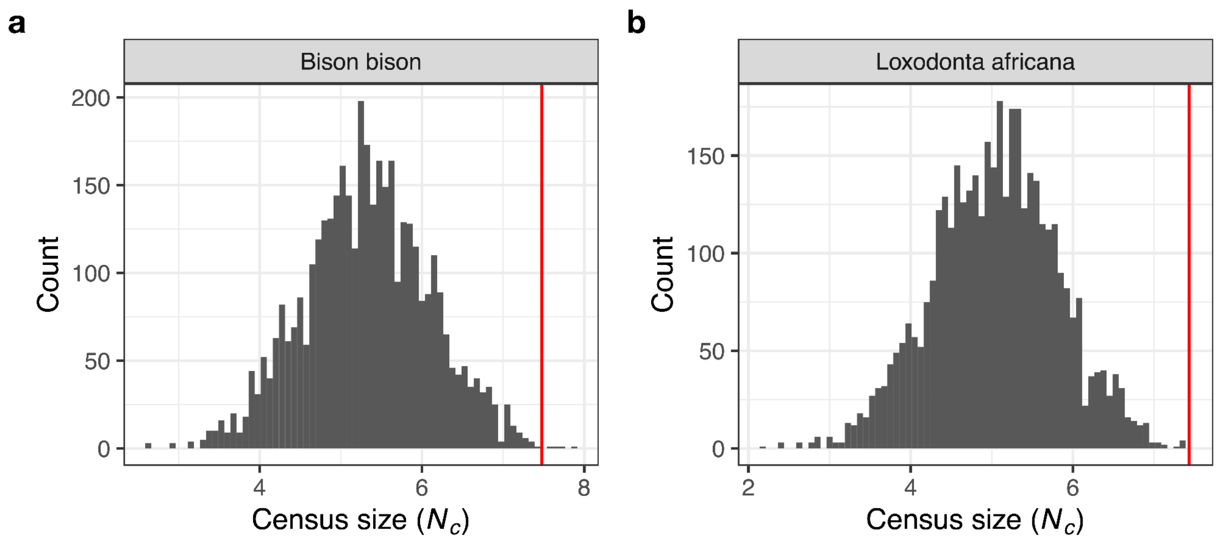

Supplementary Figure 19. Posterior sample distributions of census sizes (N_c). a Posterior sample distribution (n = 4,000) for the American bison (*Bison bison*). **b** Posterior sample distribution (n = 4,000) for the African elephant (*Loxodonta africana*). The red lines represent the historical 19th century census estimates for each species. The census size values are $\log_{10}$-transformed.

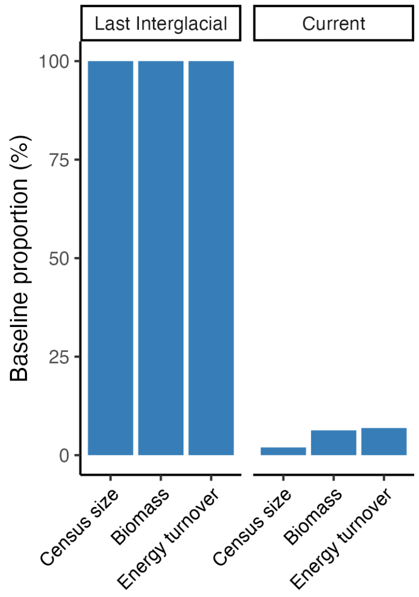

Supplementary Figure 20. Comparison of megafauna census size, biomass and energy turnover between the Last Interglacial and the current period as described in Supplementary Note 6.

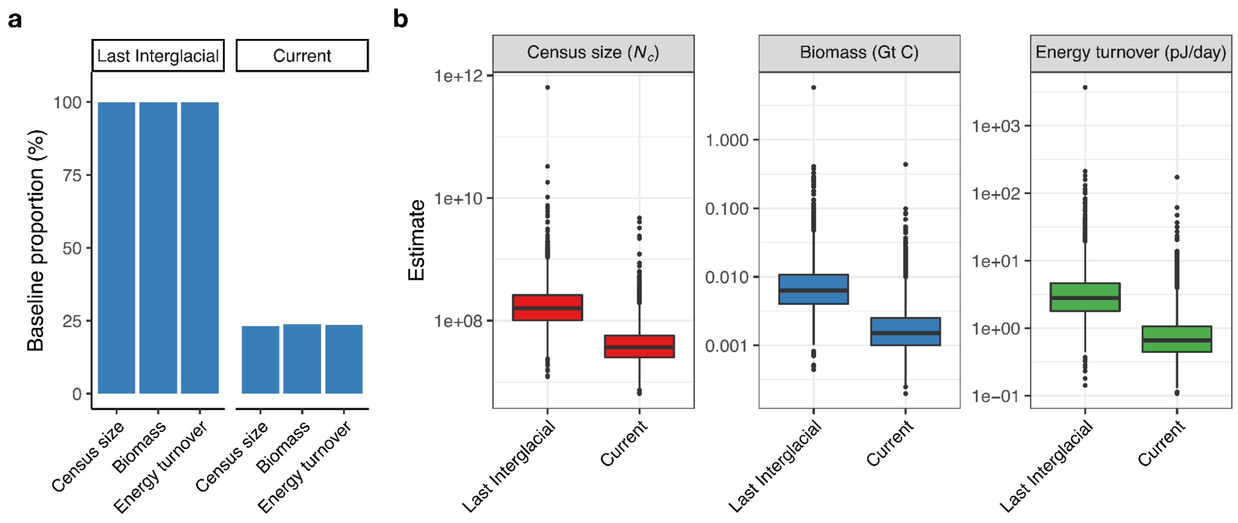

Supplementary Figure 21. Comparison of megafauna census size, biomass and energy turnover between the Last Interglacial and the current period as described in Supplementary Note 6. a Proportion of the total number of megafauna individuals (Census size), biomass and energy turnover summed across species for the current period, relative to the Last Interglacial period (calculated for the period between 116-129 kya). **b** Posterior sample distributions for the total number of megafauna individuals (Census size), biomass (measured in gigatonnes of carbon; Gt C) and energy turnover (measured in petajoules per day; pJ/day) summed across species for the Last Interglacial and current period. All distributions consist of 4,000 posterior samples (see Supplementary Note 5 and 6). The median of the distributions is represented by the horizontal line within the boxes and box edges represent the interquartile range (IQR; 25th to 75th percentile). The upper whisker extends from the upper box edge to the largest value no further than 1.5×IQR and the lower whisker extends from the lower box edge to the smallest value at most 1.5×IQR. Values outside whiskers are represented as individual points. All y-axes are $\log_{10}$-transformed.

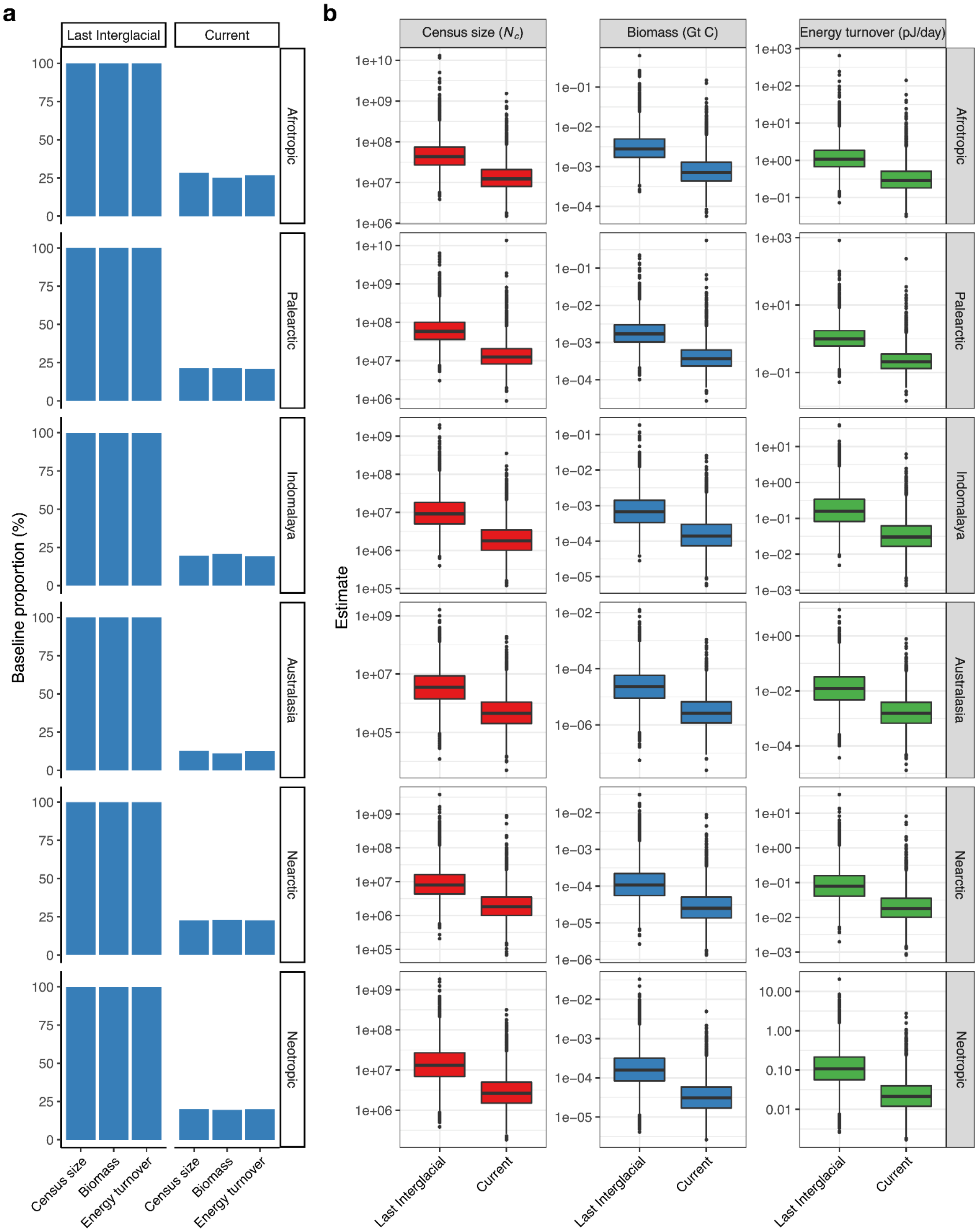

Supplementary Figure 22. Realm-specific comparison of megafauna census size, biomass and energy turnover between the Last Interglacial and the current period as described in Supplementary Note 6. a Proportion of the total number of megafauna individuals (Census size), biomass and energy turnover summed across species for the current period, relative to the Last Interglacial period (calculated for the period between 116-129 kya). **b** Posterior sample distributions for the total number of megafauna individuals (Census size), biomass (measured in gigatonnes of carbon; Gt C) and energy turnover (measured in petajoules per day; pJ/day) summed across species for the Last Interglacial and current period. All distributions consist of n = 4,000 posterior samples (see Supplementary Note 5 and 6). The median of the distributions is represented by the horizontal line within the boxes and box edges represent the interquartile range (IQR; 25th to 75th percentile). The upper whisker extends from

the upper box edge to the largest value no further than 1.5×IQR and the lower whisker extends from the lower box edge to the smallest value at most 1.5×IQR. Values outside whiskers are represented as individual points. All y-axes are log_{10}-transformed.

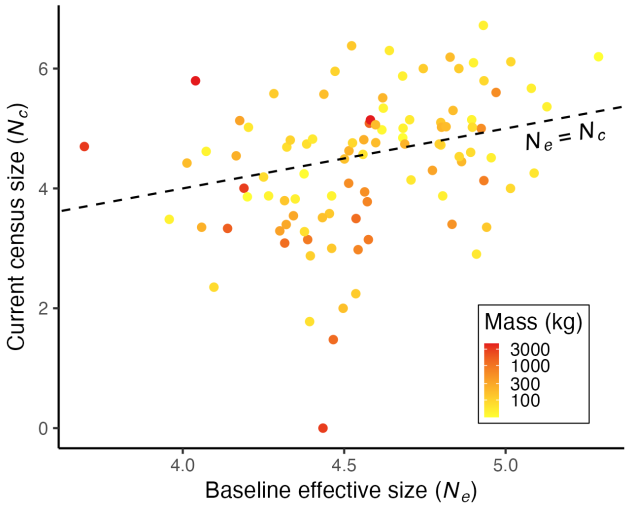

Supplementary Figure 23. Relationship between baseline effective size (N_e) calculated for the period between 100,000 to 742,719 years ago and current census size (N_c) estimated by IUCN for 99 megafauna species. Both axes are log_{10}-transformed. The dashed line is the 1:1 line.

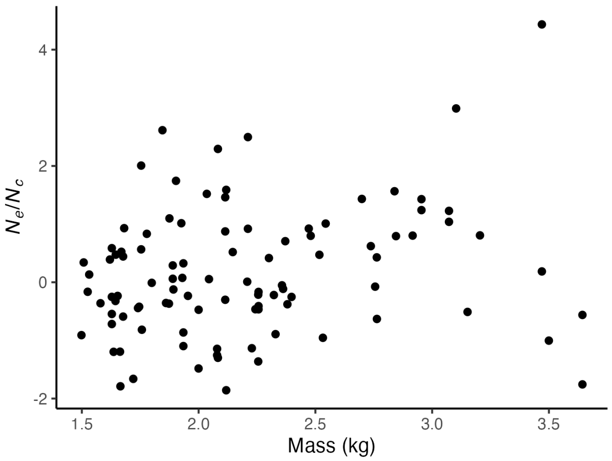

Supplementary Figure 24. Relationship between adult species mass and the N_e/N_c ratio for 99 megafauna species with available IUCN N_c estimates. N_e estimates are calculated as the average N_e for the period between 100-742 kya. Both axes are log_{10}-transformed.

Supplementary Tables

Supplementary Table 1. Piecewise linear model fit for realm-specific and global megafauna population dynamics.

Biogeographic realm	Breakpoint ± SE (in years)	Slope (N_e/year) before breakpoint [95% CI]	Slope (N_e/year) after breakpoint [95% CI]	Number of species
Afrotropic	67989.80 ± 4491.46	-1.76×10^{-8} [$-2.7\times10^{-8}0$, -0.82×10^{-8}]	-1.39×10^{-5} [-1.65×10^{-5}, -1.14×10^{-5}]	36
Palearctic	48518.58 ± 2950.23	-3.63×10^{-8} [-5.42×10^{-8}, -1.84×10^{-8}]	-2.25×10^{-5} [-2.63×10^{-5}, -1.87×10^{-5}]	32
Indomalaya	69637.21 ± 6044.86	-1.85×10^{-8} [-3.04×10^{-8}, -0.66×10^{-8}]	-1.87×10^{-5} [-2.31×10^{-5}, -1.43×10^{-5}]	11
Australasia	50559.82 ± 8071.10	6.62×10^{-8} [2.49×10^{-8}, 10.75×10^{-8}]	-2.57×10^{-5} [-3.85×10^{-5}, -1.29×10^{-5}]	4
Nearctic	35078.12 ± 3079.39	-3.25×10^{-8} [-7.04×10^{-8}, 0.55×10^{-8}]	-3.95×10^{-5} [-4.93×10^{-5}, -2.97×10^{-5}]	8
Neotropic	49425.94 ± 4783.25	0.98×10^{-8} [-1.29×10^{-8}, 3.25×10^{-8}]	-2.19×10^{-5} [-2.81×10^{-5}, -1.57×10^{-5}]	9
Global	49792.72 ± 1732.02	-1.63×10^{-8} [-2.27×10^{-8}, -1.00×10^{-8}]	-2.28×10^{-5} [-2.50×10^{-5},-2.05×10^{-5}]	100

Supplementary Table 2. Credit information for the animal photographs in Fig. 1. The photographs are under CC-BY copyright (https://creativecommons.org/licenses/by/4.0/) and adapted for the purpose of the figure.

Species	Author	Link
Antilocapra americana	opisska	https://www.inaturalist.org/observations/66825036
Elephas maximus	grihahasanov	https://www.inaturalist.org/observations/189705361
Ursus arctos	bgsmith	https://www.inaturalist.org/observations/97945564
Macropus giganteus	samuel_lee	https://www.inaturalist.org/observations/163291312
Giraffa tippelkirschi	darren_j_obbard	https://www.inaturalist.org/observations/154740030

Supplementary Table 3. Human arrival timeframes to each biogeographic realm. Arrival times (a_l depicting the lower limit of arrival time and a_u depicting the upper limit) and references are as listed in Table S1 of Andermann *et al.* (2020)[17]. In the case of Afrotropics, the timeframe of expansion of *H. sapiens* throughout the Afrotropical realm.

Biogeographic realm	a_l	a_u	Reference DOI
Afrotropics	130 kya	200 kya	-
Australasia	44 kya	65 kya	10.1038/nature22968
Indomalaya	44 kya	73 kya	10.1016/j.quascirev.2016.11.031
Nearctic	12 kya	20 kya	10.1111/ecog.01566
Neotropics	8 kya	16 kya	10.1111/ecog.01566
Palearctic	40 kya	95 kya	10.1111/ecog.01566 and 10.1038/s41559-018-0518-2

Supplementary Table 4. Piecewise linear model fit for biome-specific population dynamics.

Biome	Breakpoint ± SE	Slope before breakpoint [95% CI]	Slope after breakpoint [95% CI]	Number of species
Tropical	60056.62 ± 3547.68	-2.37×10^{-6}	-0.0048	30

		[-4.57×10^{-6}, -0.16×10^{-6}]	[-0.0056, -0.0039]	
Arid	40277.25 ± 2056.43	-5.98×10^{-6} [-8.48×10^{-6}, -3.47×10^{-6}]	-0.0063 [-0.0072, -0.0054]	42
Temperate	40212.87 ± 3363.86	-3.56v [-6.67×10^{-6}, -0.45×10^{-6}]	-0.0087 [-0.0107, -0.0066]	11
Cold	51492.88 ± 4441.01	-7.05×10^{-6} [-13.2×10^{-6}, -0.90×10^{-6}]	-0.0050 [-0.0062, -0.0038]	14
Polar	149436.78 ± 48709.36	-9.15×10^{-6} [-37.3×10^{-6}, 19.0×10^{-6}]	-0.0015 [-0.0027, -0.0003]	3

Supplementary Table 5. Piecewise linear model fit for human biogeography-specific population dynamics.

Human biogeography	Breakpoint ± Standard error	Slope before breakpoint [95% CI]	Slope after breakpoint [95% CI]	Number of species
Homo evolution	61767.04 ± 3842.07	-4.56×10^{-6} [-6.67×10^{-6}, -2.46×10^{-6}]	-0.0035 [-0.0042, -0.0029]	39
Archaic early	40382.11 ± 2146.66	-3.98×10^{-6} [-6.27×10^{-6}, -1.70×10^{-6}]	-0.0078 [-0.0090, -0.0066]	27
Archaic peripheral	70097.19 ± 18941.74	-10.7×10^{-6} [-32.4×10^{-6}, 10.9×10^{-6}]	-0.0031 [-0.0052, -0.0010]	5
Archaic late	50949.81 ± 5416.76	-10.5×10^{-6} [-17.5×10^{-6}, -3.59×10^{-6}]	-0.0047 [-0.0061, -0.0033]	8
H. sapiens only	42523.75 ± 2486.85	2.08×10^{-6} [-2.05×10^{-6}, 6.21×10^{-6}]	-0.0069 [-0.0081, -0.0058]	21

Supplementary Table 6. Realm-specific breakpoint analysis with respect to different percentage levels of species' ranges contained within the realm.

Biogeographic realm (% of species' range)	Breakpoint ± SE	Slope before breakpoint [95% CI]	Slope after breakpoint [95% CI]	Number of species
Afrotropic (>10%)	49810.97 ± 2861.96	-5.24×10^{-6} [-7.28×10^{-6}, -3.20×10^{-6}]	-0.0046 [-0.0053, -0.0038]	43
Afrotropic (>25%)	61486.18 ± 3739.89	-4.76×10^{-6} [-6.83×10^{-6}, -2.69×10^{-6}]	-0.0035 [-0.0041, -0.0029]	41
Afrotropic (>50%)	67989.79 ± 4491.46	-4.06×10^{-6} [-6.23×10^{-6}, -1.88×10^{-6}]	-0.0032 [-0.0038, -0.0026]	36
Afrotropic (>75%)	67254.25 ± 4478.59	-3.81×10^{-6} [-6.03×10^{-6}, -1.59×10^{-6}]	-0.0034 [-0.0040, -0.0027]	34
Afrotropic (>90%)	55618.42 ± 3733.69	-4.52×10^{-6} [-7.14×10^{-6}, -1.89×10^{-6}]	-0.0043 [-0.0051, -0.0035]	32
Afrotropic (>99%)	60944.48 ± 4188.98	-4.16×10^{-6} [-6.86×10^{-6}, -1.45×10^{-6}]	-0.0039 [-0.0047, -0.0031]	31
Palearctic (>10%)	51616.88 ± 3062.23	-4.32×10^{-6} [-6.49×10^{-6}, -2.15×10^{-6}]	-0.0046 [-0.0054, -0.0039]	39
Palearctic (>25%)	49910.76 ± 3130.89	-8.31×10^{-6} [-12.0×10^{-6}, -4.58×10^{-6}]	-0.0047 [-0.0056, -0.0039]	36
Palearctic (>50%)	48765.90 ± 3143.23	-8.10×10^{-6} [-12.4×10^{-6}, -3.85×10^{-6}]	-0.0052 [-0.0061, -0.0043]	30
Palearctic (>75%)	43796.50 ± 3275.49	-9.18×10^{-6} [-13.8×10^{-6}, -4.55×10^{-6}]	-0.0058 [-0.0070, -0.0046]	18

Palearctic (>90%)	50003.69 ± 4362.79	-7.88×10^{-6} [-13.0×10^{-6}, -2.72×10^{-6}]	-0.0046 [-0.0058, -0.0035]	14
Palearctic (>99%)	48765.94 ± 7216.68	-13.03×10^{-6} [-23.0×10^{-6}, -3.04×10^{-6}]	-0.0045 [-0.0063, -0.0027]	5
Indomalaya (>10%)	42702.48 ± 2567.71	-4.51×10^{-6} [-6.84×10^{-6}, -2.18×10^{-6}]	-0.0073 [-0.0085, -0.0061]	21
Indomalaya (>25%)	42884.45 ± 3056.21	-4.06×10^{-6} [-6.55×10^{-6}, -1.55×10^{-6}]	-0.0077 [-0.0092, -0.0061]	16
Indomalaya (>50%)	69637.21 ± 6044.86	-4.25×10^{-6} [-6.99×10^{-6}, -1.51×10^{-6}]	-0.0043 [-0.0053, -0.0033]	11
Indomalaya (>75%)	56316.67 ± 4647.20	-3.27×10^{-6} [-5.89×10^{-6}, -6.60×10^{-6}]	-0.0060 [-0.0074, -0.0047]	9
Indomalaya (>90%)	54739.88 ± 4861.21	-5.26×10^{-6} [-9.60×10^{-6}, -0.91×10^{-6}]	-0.0060 [-0.0075, -0.0046]	8
Indomalaya (>99%)	48308.65 ± 5552.29	-5.01×10^{-6} [-9.12×10^{-6}, -0.91×10^{-6}]	-0.0078 [-0.0102, -0.0054]	4
Australasia (>10%)	50559.82 ± 8071.10	15.2×10^{-6} [5.73×10^{-6}, 24.7×10^{-6}]	-0.0059 [-0.0089, -0.0030]	4
Australasia (>25%)	50559.82 ± 8071.10	15.2×10^{-6} [5.73×10^{-6}, 24.7×10^{-6}]	-0.0059 [-0.0089, -0.0030]	4
Australasia (>50%)	50559.82 ± 8071.10	15.2×10^{-6} [5.73×10^{-6}, 24.7×10^{-6}]	-0.0059 [-0.0089, -0.0030]	4
Australasia(>75%)	50559.82 ± 8071.10	15.2×10^{-6} [5.73×10^{-6}, 24.7×10^{-6}]	-0.0059 [-0.0089, -0.0030]	4
Australasia (>90%)	50559.82 ± 8071.10	15.2×10^{-6} [5.73×10^{-6}, 24.7×10^{-6}]	-0.0059 [-0.0089, -0.0030]	4
Australasia (>99%)	50559.82 ± 8071.10	15.2×10^{-6} [5.73×10^{-6}, 24.7×10^{-6}]	-0.0059 [-0.0089, -0.0030]	4
Nearctic (>10%)	48945.71 ± 4918.20	-6.63×10^{-6} [-15.6×10^{-6}, 2.35×10^{-6}]	-0.0058 [-0.0074, -0.0043]	13
Nearctic (>25%)	48945.71 ± 4918.20	-6.63×10^{-6} [-15.6×10^{-6}, 2.35×10^{-6}]	-0.0058 [-0.0074, -0.0043]	13
Nearctic (>50%)	35078.12 ± 3079.39	-7.47×10^{-6} [-16.2×10^{-6}, 1.26×10^{-6}]	-0.0091 [-0.0114, -0.0068]	8
Nearctic (>75%)	32942.81 ± 2545.21	-15.7×10^{-6} [-25.0×10^{-6}, -5.49×10^{-6}]	-0.0090 [-0.0110, -0.0070]	7
Nearctic (>90%)	32942.81 ± 2545.21	-15.7×10^{-6} [-25.0×10^{-6}, -5.49×10^{-6}]	-0.0090 [-0.0110, -0.0070]	7
Nearctic (>99%)	30493.37 ± 3307.31	-14.7×10^{-6} [-25.1×10^{-6}, -4.40×10^{-6}]	-0.0095 [-0.0124, -0.0065]	6
Neotropic (>10%)	51137.84 ± 4997.7	3.78×10^{-6} [-1.37×10^{-6}, 8.93×10^{-6}]	-0.0051 [-0.0066, -0.0036]	10
Neotropic (>25%)	51137.84 ± 4997.7	3.78×10^{-6} [-1.37×10^{-6}, 8.93×10^{-6}]	-0.0051 [-0.0066, -0.0036]	10
Neotropic (>50%)	49425.95 ± 4783.25	2.26×10^{-6} [-2.97×10^{-6}, 7.49×10^{-6}]	-0.0050 [-0.0065, -0.0036]	9
Neotropic (>75%)	54025.06 ± 7103.83	2.03×10^{-6} [-3.39×10^{-6}, 7.45×10^{-6}]	-0.0040 [-0.0056, -0.0025]	8
Neotropic (>90%)	54025.06 ± 7103.83	2.03×10^{-6} [-3.39×10^{-6}, 7.45×10^{-6}]	-0.0040 [-0.0056, -0.0025]	8
Neotropic (>99%)	55460.05 ± 8206.94	2.81×10^{-6} [-2.89×10^{-6}, 8.51×10^{-6}]	-0.0041 [-0.0059, -0.0023]	7

Supplementary Table 7. Biome-specific breakpoint analysis with respect to different percentage levels of species' ranges contained within the biome.

Biogeographic realm (% of species' range)	Breakpoint ± SE	Slope before breakpoint [95% CI]	Slope after breakpoint [95% CI]	Number of species
Tropical (>10%)	55025.30 ± 2591.59	-3.06×10^{-6} [-4.62×10^{-6}, -1.49×10^{-6}]	-0.0045 [-0.0051, -0.0039]	57
Tropical (>25%)	61486.18 ± 3599.64	-3.03×10^{-6} [-4.66×10^{-6}, -1.41×10^{-6}]	-0.0039 [-0.0046, -0.0033]	42

Tropical (>50%)	60629.89 ± 3819.17	-2.40×10^{-6} [-4.87×10^{-6}, 0.07×10^{-6}]	-0.0046 [-0.0054, -0.0037]	24
Tropical (>75%)	60111.97 ± 4410.50	-2.74×10^{-6} [-5.52×10^{-6}, 0.05×10^{-6}]	-0.0054 [-0.0065, -0.0042]	15
Tropical (>90%)	66808.27 ± 7054.66	-4.76×10^{-6} [-7.88×10^{-6}, -1.64×10^{-6}]	-0.0039 [-0.0051, -0.0027]	10
Tropical (>99%)	93112.27 ± 12618.34	-8.15×10^{-6} [-11.6×10^{-6}, -4.69×10^{-6}]	-0.0024 [-0.0033, -0.0015]	5
Arid (>10%)	45045.24 ± 1804.49	-5.66×10^{-6} [-7.73×10^{-6}, -3.59×10^{-6}]	-0.0059 [-0.0065, -0.0052]	72
Arid (>25%)	44982.79 ± 2125.55	-4.63×10^{-6} [-6.71×10^{-6}, -2.56×10^{-6}]	-0.0053 [-0.0060, -0.0046]	55
Arid (>50%)	39123.19 ± 2287.47	-7.81×10^{-6} [-11.1×10^{-6}, -4.49×10^{-6}]	-0.0065 [-0.0076, -0.0054]	33
Arid (>75%)	29818.75 ± 2175.34	-8.83×10^{-6} [-15.0×10^{-6}, -2.64×10^{-6}]	-0.0103 [-0.0125, -0.0081]	16
Arid (>90%)	30583.30 ± 3070.23	-2.49×10^{-6} [-10.9×10^{-6}, 5.91×10^{-6}]	-0.0084 [-0.0109, -0.0058]	6
Arid (>99%)	45215.25 ± 14313.67	-13.1×10^{-6} [-25.5×10^{-6}, -0.87×10^{-6}]	-0.0027 [-0.0052, -0.0002]	2
Temperate (>10%)	52923.64 ± 2233.40	-2.25×10^{-6} [-3.94×10^{-6}, -0.55×10^{-6}]	-0.0050 [-0.0056, -0.0044]	58
Temperate (>25%)	49924.22 ± 3459.73	-2.70×10^{-6} [-5.35×10^{-6}, -0.04×10^{-6}]	-0.0057 [-0.0069, -0.0046]	21
Temperate (>50%)	40058.89 ± 3566.48	-6.66×10^{-6} [-16.3×10^{-6}, 3.02×10^{-6}]	-0.0096 [-0.0120, -0.0072]	5
Temperate (>75%)	41036.33 ± 6708.53	-5.12×10^{-6} [-13.5×10^{-6}, 3.28×10^{-6}]	-0.0087 [-0.0135, -0.0039]	1
Temperate (>90%)	NA	NA	NA	0
Temperate (>99%)	NA	NA	NA	0
Cold (>10%)	42889.53 ± 2346.92	-7.57×10^{-6} [-11.8×10^{-6}, -3.32×10^{-6}]	-0.0066 [-0.0077, -0.0057]	32
Cold (>25%)	48765.90 ± 3646.03	-6.26×10^{-6} [-11.9×10^{-6}, -0.62×10^{-6}]	-0.0056 [-0.0067, -0.0045]	22
Cold (>50%)	53653.29 ± 4792.63	-9.65×10^{-6} [-16.5×10^{-6}, -2.79×10^{-6}]	-0.0047 [-0.0058, -0.0036]	11
Cold (>75%)	NA	NA	NA	0
Cold (>90%)	NA	NA	NA	0
Cold (>99%)	NA	NA	NA	0
Polar (>10%)	53653.28 ± 5257.16	-11.1×10^{-6} [-19.0×10^{-6}, -3.25×10^{-6}]	-0.0046 [-0.0058, -0.0034]	17
Polar (>25%)	49910.75 ± 8392.59	-16.8×10^{-6} [-29.1×10^{-6}, -4.40×10^{-6}]	-0.0043 [-0.0062, -0.0024]	10
Polar (>50%)	2010.10 ± 260.61	-548.3×10^{-6} [-879.3×10^{-6}, -217.3×10^{-6}]	0.7613 [0.3729, 1.148]	1
Polar (>75%)	NA	NA	NA	0
Polar (>90%)	NA	NA	NA	0
Polar (>99%)	NA	NA	NA	0

Supplementary Table 8. Human biogeography-specific breakpoint analysis with respect to different percentage levels of species' ranges contained within the human biogeography region.

Biogeographic realm (% of species' range)	Breakpoint ± SE	Slope before breakpoint [95% CI]	Slope after breakpoint [95% CI]	Number of species

Homo evolution (>10%)	49810.97 ± 2861.96	-5.24×10^{-6} [-7.28×10^{-6}, -3.20×10^{-6}]	-0.0046 [-0.0053, -0.0038]	43
Homo evolution (>25%)	49810.97 ± 2861.96	-5.24×10^{-6} [-7.28×10^{-6}, -3.20×10^{-6}]	-0.0046 [-0.0053, -0.0038]	43
Homo evolution (>50%)	61912.31 ± 4014.05	-4.42×10^{-6} [-6.54×10^{-6}, -2.30×10^{-6}]	-0.0035 [-0.0041, -0.0029]	38
Homo evolution (>75%)	67254.25 ± 4478.59	-3.81×10^{-6} [-6.03×10^{-6}, -1.59×10^{-6}]	-0.0034 [-0.0040, -0.0027]	34
Homo evolution (>90%)	61486.19 ± 4231.41	-4.76×10^{-6} [-7.26×10^{-6}, -2.26×10^{-6}]	-0.0037 [-0.0045, -0.0030]	33
Homo evolution (>99%)	55618.42 ± 3733.69	-4.51×10^{-6} [-7.14×10^{-6}, -1.89×10^{-6}]	-0.0043 [-0.0052, -0.0035]	32
Archaic early (>10%)	49981.48 ± 2513.19	-3.95×10^{-6} [-5.91×10^{-6}, -2.00×10^{-6}]	-0.0055 [-0.0062, -0.0047]	37
Archaic early (>25%)	42569.64 ± 2237.90	-4.49×10^{-6} [-6.66×10^{-6}, -2.31×10^{-6}]	-0.0066 [-0.0076, -0.0056]	32
Archaic early (>50%)	42113.33 ± 2338.31	-3.93×10^{-6} [-6.25×10^{-6}, -1.61×10^{-6}]	-0.0075 [-0.0087, -0.0063]	26
Archaic early (>75%)	53612.97 ± 3924.00	-3.63×10^{-6} [-6.22×10^{-6}, -1.04×10^{-6}]	-0.0060 [-0.0072, -0.0048]	16
Archaic early (>90%)	54186.39 ± 4258.88	-5.57×10^{-6} [-9.00×10^{-6}, -2.13×10^{-6}]	-0.0057 [-0.0070, -0.0045]	12
Archaic early (>99%)	47866.49 ± 3720.05	-4.12×10^{-6} [-7.26×10^{-6}, -0.98×10^{-6}]	-0.0077 [-0.0093, -0.0060]	8
Archaic peripheral (>10%)	55002.595 ± 7984.10	-5.68×10^{-6} [-16.7×10^{-6}, 5.64×10^{-6}]	-0.0044 [-0.0060, -0.0027]	9
Archaic peripheral (>25%)	70097.229 ± 18941.76	-10.7×10^{-6} [-32.4×10^{-6}, 10.9×10^{-6}]	-0.0031 [-0.0052, -0.0010]	5
Archaic peripheral (>50%)	6899.918 ± 2144.31	-187.8×10^{-6} [-263.0×10^{-6}, -112.6×10^{-6}]	-0.0283 [-0.0557, -0.0009]	1
Archaic peripheral (>75%)	6899.918 ± 2144.31	-187.8×10^{-6} [-263.0×10^{-6}, -112.6×10^{-6}]	-0.0283 [-0.0557, -0.0009]	1
Archaic peripheral (>90%)	6899.918 ± 2144.31	-187.8×10^{-6} [-263.0×10^{-6}, -112.6×10^{-6}]	-0.0283 [-0.0557, -0.0009]	1
Archaic peripheral (>99%)	NA	NA	NA	0
Archaic late (>10%)	49910.76 ± 3331.93	-2.95×10^{-6} [-5.73×10^{-6}, -0.16×10^{-6}]	-0.0058 [-0.0069, -0.0047]	21
Archaic late (>25%)	48890.35 ± 3847.66	-10.8×10^{-6} [-16.2×10^{-6}, -5.34×10^{-6}]	-0.0054 [-0.0065, -0.0042]	14
Archaic late (>50%)	48319.34 ± 5831.75	-11.1×10^{-6} [-18.8×10^{-6}, -3.39×10^{-6}]	-0.0049 [-0.0066, -0.0033]	7
Archaic late (>75%)	48319.34 ± 5831.75	-11.1×10^{-6} [-18.8×10^{-6}, -3.39×10^{-6}]	-0.0049 [-0.0066, -0.0033]	7
Archaic late (>90%)	37662.57 ± 5655.94	-12.1×10^{-6} [-21.1×10^{-6}, -2.98×10^{-6}]	-0.0061 [-0.0088, -0.0033]	4
Archaic late (>99%)	NA	NA	NA	0
H. sapiens only (>10%)	49113.36 ± 2863.88	1.06×10^{-6} [-2.76×10^{-6}, 4.89×10^{-6}]	-0.0057 [-0.0067, -0.0048]	2
H. sapiens only (>25%)	47431.59 ± 2862.96	0.74×10^{-6} [-3.23×10^{-6}, 4.71×10^{-6}]	-0.0060 [-0.0070, -0.0049]	21
H. sapiens only (>50%)	42770.36 ± 2565.31	0.07×10^{-6} [-3.36×10^{-6}, 4.80×10^{-6}]	-0.0068 [-0.0080, -0.0056]	20
H. sapiens only (>75%)	41896.21 ± 2937.86	2.54×10^{-6} [-1.93×10^{-6}, 7.01×10^{-6}]	-0.0067 [-0.0081, -0.0053]	14
H. sapiens only (>90%)	60183.96 ± 5989.10	3.77×10^{-6} [-0.92×10^{-6}, 8.46×10^{-6}]	-0.0038 [-0.0049, -0.0027]	11

H. sapiens only (>99%)	60183.96 ± 5989.10	3.77×10^{-6} [-0.92×10^{-6}, 8.46×10^{-6}]	-0.0038 [-0.0049, -0.0027]	11

Supplementary Table 9. 67 megafauna species chosen as representatives of their genus.

Representative species
Acinonyx jubatus
Addax nasomaculatus
Aepyceros melampus
Ailuropoda melanoleuca
Alces alces gigas
Ammotragus lervia
Antidorcas marsupialis
Antilocapra americana
Axis porcinus
Babyrousa celebensis
Beatragus hunteri
Bison bison
Bos mutus
Bubalus bubalis
Budorcas taxicolor
Camelus ferus
Capra ibex
Catagonus wagneri
Ceratotherium simum simum
Cervus canadensis
Connochaetes taurinus
Crocuta crocuta
Dicerorhinus sumatrensis sumatrensis
Diceros bicornis
Elaphurus davidianus
Elephas maximus
Equus hemionus
Giraffa tippelskirchi
Gorilla gorilla gorilla
Helarctos malayanus
Hemitragus hylocrius
Hexaprotodon liberiensis
Hippopotamus amphibius
Hippotragus niger niger
Hyaena hyaena
Hydrochoerus hydrochaeris
Kobus ellipsiprymnus
Lama guanicoe guanicoe
Litocranius walleri
Loxodonta africana
Macropus rufus
Myrmecophaga tridactyla
Nanger granti
Odocoileus virginianus
Okapia johnstoni
Oreamnos americanus
Orycteropus afer
Oryx dammah
Ovibos moschatus
Ovis vignei
Pan troglodytes troglodytes
Panthera leo

Parahyaena brunnea
Phacochoerus africanus
Pongo abelii
Pseudois nayaur
Puma concolor
Rangifer tarandus caribou
Redunca redunca
Rhinoceros unicornis
Sus scrofa scrofa
Syncerus caffer
Tapirus indicus
Tragelaphus oryx
Tremarctos ornatus
Ursus americanus
Vicugna vicugna vicugna

Supplementary Table 10. Climate-based, human-based and combined explanatory models of population size.

Abbreviation	Class	Type
linT	Climate-only	Linear effect of temperature
quadT	Climate-only	Quadratic effect of temperature
linT+L	Climate-only	Linear effect of temperature and temperature lag
quadT+L	Climate-only	Quadratic effect of temperature and temperature lag
linP	Climate-only	Linear effect of precipitation
quadP	Climate-only	Quadratic effect of precipitation
linP+L	Climate-only	Linear effect of precipitation and precipitation lag
quadP+L	Climate-only	Quadratic effect of precipitation and precipitation lag
linT+linP	Climate-only	Linear effect of temperature and precipitation
quadT+quadP	Climate-only	Quadratic effect of temperature and precipitation
linT+linP+L	Climate-only	Linear effect of temperature, precipitation, temperature lag and precipitation lag
quadT+quadP+L	Climate-only	Quadratic effect of temperature, precipitation, temperature lag and precipitation lag
pH	Human-only	Linear effect of probability of human presence

linH	Human-only	Linear effect of human impact post arrival
expH	Human-only	Exponential effect of human impact post arrival
logH	Human-only	Logistic effect of human impact post arrival
quadT+L+pH	Combined	Combination of effects as described above
quadT+L+linH	Combined	Combination of effects as described above
quadT+L+expH	Combined	Combination of effects as described above
quadT+L+logH	Combined	Combination of effects as described above
linT+linP+L+pH	Combined	Combination of effects as described above
linT+linP+L+linH	Combined	Combination of effects as described above
linT+linP+L+expH	Combined	Combination of effects as described above
linT+linP+L+logH	Combined	Combination of effects as described above
linT+L+pH	Combined	Combination of effects as described above
linT+L+linH	Combined	Combination of effects as described above
linT+L+expH	Combined	Combination of effects as described above
linT+L+logH	Combined	Combination of effects as described above
quadT+pH	Combined	Combination of effects as described above
quadT+linH	Combined	Combination of effects as described above
quadT+expH	Combined	Combination of effects as described above
quadT+logH	Combined	Combination of effects as described above

Supplementary Table 11. Example for the calculation of the probability of human presence p_H.

a_l	a_u	t_l	t_u	p
40 kya	95 kya	100 kya	125 kya	0
40 kya	95 kya	75 kya	100 kya	0.36
40 kya	95 kya	50 kya	75 kya	0.81
40 kya	95 kya	25 kya	50 kya	1
40 kya	95 kya	0 kya	25 kya	1

Supplementary Table 12. Summary statistics for the distributions of the sums of census size, biomass and energy turnover during the baseline period (100-742 kya; Supplementary Fig. 18b) across different megafauna species categories.

Species category	Value category	Census size ($\times 10^9$)	Biomass (Gt C)	Energy turnover (pJ/day)
Extant; included	50th-percentile	0.152	0.007	2.758
Extant; missing	50th-percentile	0.156	0.002	1.137
Extinct	50th-percentile	0.245	0.023	5.860
ALL*	50th-percentile	0.600	0.034	10.877
Extant; included	95th-percentile	0.609	0.029	11.450
Extant; missing	95th-percentile	0.351	0.005	2.826
Extinct	95th-percentile	0.510	0.071	16.351
ALL	95th-percentile	1.263	0.094	27.044
Extant; included	99th-percentile	1.753	0.077	32.133
Extant; missing	99th-percentile	0.585	0.010	4.885
Extinct	99th-percentile	0.873	0.143	28.841
SUM	99th-percentile	2.384	0.177	52.093
Extant; included	MAX†	15.741	1.042	403.705
Extant; missing	MAX	1.689	0.033	18.726
Extinct	MAX	2.655	0.653	99.623
ALL	MAX	16.431	1.067	411.954
ALL	Theoretical MAX‡	20.085	1.727	522.054

*The distribution for all species was calculated by summing randomly sampled values (without replacement) from each of the distributions of the three species categories.

†Maximum value of a distribution.

‡Maximum possible value of a distribution for all species calculated as the sum of the maximum values from each of the distributions of the three species categories.

Supplementary Table 13. Response and explanatory variables used for modelling (Supplementary Note 1).

Variable	Type	Description
M	Response	Per generation mutation rate
G	Explanatory	Generation time
N_e	Response, explanatory	Average effective population size across the focal time interval
t	Explanatory	Mid time-point of focal time interval or time since human arrival
m	Explanatory	Adult mass
D	Response	Decline severity
t^{MIN}	Explanatory	Mid time-point of time interval when a species achieved the lowest population size
t^{MAX}	Explanatory	Mid time-point of time interval when a species achieved the highest population size
T	Explanatory	Average temperature of the focal time interval
P	Explanatory	Average precipitation of the focal time interval
L	Explanatory	Average temperature of the preceding time interval (temperature lag)
p	Explanatory	Probability of human presence
H	Explanatory	Indicator for overlap of time interval with human arrival range
N_c	Response, explanatory	Average census population size across the focal time interval

References

1. Li, H. & Durbin, R. Inference of human population history from individual whole-genome sequences. *Nature* **475**, 493–496 (2011).

2. Bergeron, L. A. *et al.* Evolution of the germline mutation rate across vertebrates. *Nature* **615**, 285–291 (2023).

3. Andermann, T., Faurby, S., Turvey, S. T., Antonelli, A. & Silvestro, D. The past and future human impact on mammalian diversity. *Sci Adv* **6**, (2020).

4. Faurby, S. *et al.* PHYLACINE 1.2: The Phylogenetic Atlas of Mammal Macroecology. *Ecology* **99**, 2626 (2018).

5. Pedersen, R. Ø., Faurby, S. & Svenning, J.-C. Late‐Quaternary megafauna extinctions have strongly reduced mammalian vegetation consumption. *Glob. Ecol. Biogeogr.* (2023) doi:10.1111/geb.13723.

6. Isenberg, A. C. *The Destruction of the Bison: An Environmental History, 1750-1920*. (Cambridge University Press, 2000).

7. Milner-Gulland, E. J. & Beddington, J. R. The exploitation of elephants for the ivory trade: an historical perspective. *Proc. Biol. Sci.* **252**, 29–37 (1993).

8. Muggeo, V. M. R. Estimating regression models with unknown break-points. *Stat. Med.* **22**, 3055–3071 (2003).

9. Hadfield, J. D. MCMC methods for multi-response generalized linear mixed models: TheMCMCglmmRPackage. *J. Stat. Softw.* **33**, (2010).

10. Pagel, M. Inferring the historical patterns of biological evolution. *Nature* **401**, 877–884 (1999).

11. Chen, L. *et al.* Large-scale ruminant genome sequencing provides insights into their evolution and distinct traits. *Science* **364**, (2019).

12. Schaffner, S. F. *et al.* Calibrating a coalescent simulation of human genome sequence variation. *Genome Res.* **15**, 1576–1583 (2005).

13. 1000 Genomes Project Consortium *et al.* A global reference for human genetic variation. *Nature* **526**,

68–74 (2015).

14. Bergström, A., Stringer, C., Hajdinjak, M., Scerri, E. M. L. & Skoglund, P. Origins of modern human ancestry. *Nature* **590**, 229–237 (2021).

15. Pedersen, R. Ø., Faurby, S. & Svenning, J.-C. Extinctions have strongly reduced the mammalian consumption of primary productivity. (2020) doi:10.1101/2020.10.15.341297.

16. Malhi, Y. *et al.* Megafauna and ecosystem function from the Pleistocene to the Anthropocene. *Proc. Natl. Acad. Sci. U. S. A.* **113**, 838–846 (2016).

17. Hoffman, J. S., Clark, P. U., Parnell, A. C. & He, F. Regional and global sea-surface temperatures during the last interglaciation. *Science* **355**, 276–279 (2017).

18. Otto-Bliesner, B. L. *et al.* How warm was the last interglacial? New model-data comparisons. *Philos. Trans. A Math. Phys. Eng. Sci.* **371**, 20130097 (2013).
