# Supplementary material for: Worldwide Late Pleistocene and Early Holocene population declines in extant megafauna are associated with Homo sapiens expansion rather than climate change
Source: Nat Commun. 2023 Nov 24;14:7679. doi: 10.1038/s41467-023-43426-5 (PMC10667484; doi:10.1038/s41467-023-43426-5)
Supplement: Supplementary file 3 — Description of Additional Supplementary Files [file 41467_2023_43426_MOESM3_ESM.pdf]

Description of Additional Supplementary Files

File Name: Supplementary Data 1
Description: List of all species used in the study including estimates of species' adult mass, generation time, mutation rate, and accession codes for reference genome sequences and short-read data.

File Name: Supplementary Data 2
Description: List of species with classification with respect to biogeographic realm, biome and human biogeography.

File Name: Supplementary Data 3
Description: PSMC trajectories of species.

File Name: Source Data 1
Description: PSMC trajectories of species for Fig. 1a.

File Name: Source Data 2
Description: Piecewise linear model fit for Fig. 1a.

File Name: Source Data 3
Description: Slope values for Fig 1b.

File Name: Source Data 4
Description: Decline severity values for Fig 1c.

File Name: Source Data 5
Description: PSMC trajectories of species for the last 742 ky for Fig. 2a.

File Name: Source Data 6
Description: Average population size of species before and after 100 kya for Fig. 2a.

File Name: Source Data 7
Description: Temperature values for the last 742 ky for Fig. 2a.

File Name: Source Data 8
Description: Observed and predicted population size values - climate-only model for Fig. 2b.

File Name: Source Data 9
Description: Mean squared error (MSE) - climate-only model for Fig. 2b.

File Name: Source Data 10
Description: Log-score model values for Fig. 3a.

File Name: Source Data 11
Description: Mean squared error (MSE) - all models for Fig. 3b.

File Name: Source Data 12
Description: Per-species mean squared errors (MSE) - all models for Fig. 3c.

File Name: Source Data 13
Description: Megafauna parameters - 139 species for Fig. 4a.

File Name: Source Data 14
Description: Megafauna parameters - all species for Fig. 4b.
